# Mitochondrial Quality Control Impairment Is a Hallmark of TDP-43^G376D^ ALS Patient-Derived Fibroblasts

**DOI:** 10.3390/antiox15091051

**Published:** 2026-08-22

**Authors:** Giuseppe Petito, Maria Ventriglia, Victoria Stefania Del Fiore, Arianna Cuomo, Federica Cioffi, Francesco Manfrevola, Flora Guerra, Lucia Bertuccini, Giulia Ricci, Gilda Cobellis, Antonia Lanni, Cecilia Bucci, Roberta Romano, Rosalba Senese

**Affiliations:** 1 Department of Environmental, Biological and Pharmaceutical Sciences and Technologies, University of Campania “L. Vanvitelli”, Via Vivaldi 43, 81100 Caserta, Italy; giuseppe.petito@unicampania.it (G.P.); maria.ventriglia@unicampania.it (M.V.); arianna.cuomo@unicampania.it (A.C.); antonia.lanni@unicampania.it (A.L.); 2 Department of Experimental Medicine (DiMeS), University of Salento, Via Provinciale Lecce-Monteroni n.165, 73100 Lecce, Italy; victoriastefania.delfiore@unisalento.it (V.S.D.F.); cecilia.bucci@unisalento.it (C.B.); 3 Department of Sciences and Technologies, University of Sannio, 82100 Benevento, Italy; federica.cioffi@unisannio.it; 4 Department of Experimental Medicine, University of Campania “L. Vanvitelli”, 80138 Naples, Italy; francesco.manfrevola@unicampania.it (F.M.); giulia.ricci@unicampania.it (G.R.); gilda.cobellis@unicampania.it (G.C.); 5 Department of Biological and Environmental Sciences and Technologies (DiSTeBA), University of Salento, Via Provinciale Lecce-Monteroni n.165, 73100 Lecce, Italy; flora.guerra@unisalento.it; 6 Core Facilities Microscopy Area, Italian National Institute of Health, Viale Regina Elena n. 299, 00161 Rome, Italy; lucia.bertuccini@iss.it

**Keywords:** Amyotrophic Lateral Sclerosis (ALS), TDP-43, mitochondrial dysfunction, Mitochondrial Quality Control (MQC), mitophagy, mitochondrial Unfolded Protein Response (UPR^mt^), oxidative stress, Endoplasmic Reticulum stress

## Abstract

Amyotrophic Lateral Sclerosis (ALS) is a progressive neurodegenerative disorder strongly associated with mitochondrial dysfunction and impaired proteostasis. Mutations in TARDBP, encoding TAR DNA-binding protein 43 (TDP-43), contribute to disease pathogenesis through cytoplasmic mislocalization and aggregation. Among these, the ALS-linked TDP-43^G376D^ mutation has been previously associated with oxidative stress, mitochondrial fragmentation, and impaired oxidative phosphorylation. Here, we investigated the impact of TDP-43^G376D^ on Mitochondrial Quality Control (MQC) pathways using patient-derived dermal fibroblasts carrying the mutation at early and advanced disease stages, complemented by HEK293T and Neuro2a cellular models expressing mutant TDP-43. We show that TDP-43^G376D^ impairs mitophagic flux, as evidenced by reduced delivery of damaged mitochondria to lysosomes. This was accompanied by pronounced disruption of mitochondrial cristae architecture and accumulation of mitochondrial DNA damage, indicating compromised mitochondrial genome integrity. Furthermore, TDP-43^G376D^ induces sustained activation of the mitochondrial Unfolded Protein Response (UPR^mt^), consistent with persistent mitochondrial stress, while selectively impairing the sirtuin-dependent antioxidant branch. In parallel, activation of the Endoplasmic Reticulum UPR (UPR^ER^) was observed, indicating a coordinated engagement of cellular stress pathways. Collectively, our findings identify coordinated alterations in multiple MQC pathways associated with TDP-43^G376D^ rather than isolated mitochondrial defects, supporting further investigation of these pathways in larger and disease-relevant ALS models.

## 1. Introduction

Mitochondria play a central role in cellular homeostasis, regulating energy production, calcium metabolism, redox balance, and intrinsic cell death pathways [1]. Given these critical functions, mitochondrial alterations are closely associated with the onset and progression of several disorders, particularly neurodegenerative diseases [2]. Disruption of mitochondrial homeostasis leads to excessive production of free radicals, release of pro-apoptotic factors, and impairment of proteostatic and autophagic machinery due to the accumulation of dysfunctional mitochondria, thereby contributing to cellular toxicity and death [3]. Moreover, excessive production of Reactive Oxygen Species (ROS) directly affects mitochondrial DNA (mtDNA) leading to oxidative damage that further impairs mitochondrial function [4].

To preserve their function, mitochondria rely on Mitochondrial Quality Control (MQC) mechanisms, which coordinate the removal of damaged proteins and dysfunctional organelles. Rather than acting as isolated processes, MQC pathways operate as a highly interconnected network that integrates proteostasis, mitophagy, and structural remodeling to preserve mitochondrial function under stress conditions [5]. When misfolded or unfolded proteins accumulate within the mitochondrial matrix, a specific stress response termed the mitochondrial Unfolded Protein Response (UPR^mt^) is activated [6,7]. In mammals, the canonical UPR^mt^ pathway depends on the coordinated activity of transcription factors including Activating Transcription Factor 4 (ATF-4), ATF-5, and C/EBP Homologous Protein (CHOP) [8,9,10]. Upon activation, these factors translocate to the nucleus and induce the expression of genes involved in mitochondrial proteostasis [10,11,12,13]. Although initially adaptive, sustained UPR^mt^ activation under chronic stress conditions may reflect persistent mitochondrial dysfunction and contribute to impaired cellular homeostasis [14,15,16]. Importantly, UPR^mt^ signaling functionally intersects with other stress pathways, including the Endoplasmic Reticulum UPR (UPR^ER^), highlighting coordinated inter-organelle stress responses [13,17]. Beyond the canonical pathway, additional UPR^mt^ axes contribute to mitochondrial homeostasis [18]. Among them, the sirtuin UPR^mt^ axis supports mitochondrial redox balance by promoting the expression of antioxidant defenses, thereby limiting oxidative stress-induced damage [18,19]. When damaged mitochondria cannot be repaired, they are selectively removed through mitophagy, a process also involved in basal mitochondrial turnover [20]. Defects in mitophagic flux, rather than in its initiation, have been increasingly associated with the accumulation of dysfunctional mitochondria and disease progression [21,22,23].

An additional key component of MQC is the structural organization of mitochondrial cristae. Cristae architecture is regulated by the mitochondrial contact site and cristae organizing system (MICOS), a multiproteic complex responsible for maintaining inner membrane organization [24]. Emerging evidence indicates that cristae remodeling is tightly linked to mitochondrial stress responses and MQC pathways, and its disruption impairs oxidative phosphorylation efficiency and cellular adaptation to stress [25,26].

In neurodegenerative diseases, mitochondrial dysfunction, oxidative stress, and defective proteostasis are tightly interconnected [27,28,29]. Among these disorders, Amyotrophic Lateral Sclerosis (ALS) is characterized by progressive degeneration of motor neurons in the spinal cord, brainstem, and motor cortex [30]. TARDBP encodes TAR DNA-binding protein 43 (TDP-43), a nuclear protein involved in RNA metabolism [31]. ALS-associated mutations lead to its cytoplasmic mislocalization and aggregation, representing a major hallmark of the disease [32]. We recently characterized the G376D mutation in TDP-43, which induces protein mislocalization, cytoplasmic aggregation, and mitochondrial accumulation [33,34]. Furthermore, TDP-43^G376D^ is associated with lysosomal and mitochondrial dysfunction, including impaired mitochondrial biogenesis, defective oxidative phosphorylation, enhanced mitochondrial fragmentation, and increased oxidative stress [35,36].

However, it remains unclear whether TDP-43^G376D^-associated mitochondrial dysfunction arises from isolated defects or reflects a broader impairment of MQC networks. In particular, the extent to which multiple MQC pathways are concurrently affected by the TDP-43^G376D^ mutation remains poorly understood. Since MQC pathways operate as an integrated functional network, impairment of one of these components may propagate throughout the system, ultimately resulting in global mitochondrial failure. To address this question, we investigated the impact of the TDP-43^G376D^ mutation using both cellular and patient-derived models, including HEK293T and Neuro2a cells expressing mutant TDP-43, as well as dermal fibroblasts obtained from skin biopsies of ALS patients carrying the G376D mutation. We analyzed key MQC mechanisms, with a specific focus on mitophagy, mitochondrial cristae architecture, mtDNA integrity, and activation of the UPR^mt^, as well as its functional interplay with the UPR^ER^ pathway.

## 2. Materials and Methods

### 2.1. Cell Lines and Patient-Derived Dermal Fibroblasts

Primary dermal fibroblasts were established from skin biopsy specimens obtained from two age- and sex-matched healthy male donors and two male patients with ALS. These patients belong to different branches of the same large Italian family with familial ALS associated with the heterozygous *TARDBP* c.1127G>A (p.G376D) variant, originally identified in a patient from Southern Italy by Conforti et al. [37]. Genetic analysis on several members of this family was performed by Sanger sequencing of the *TARDBP* coding exons and flanking intronic regions, and the variant was classified as likely pathogenic according to ACMG criteria [38].

Fibroblast lines used in this study were derived from skin biopsies performed on two patients at advanced symptomatic stages, hereafter referred to as ALS1A and ALS2A (A = advanced). In addition, fibroblasts named ALS1O (O = onset) were derived from the same patient (Patient 1) during the early stage of disease progression. Skin biopsies from Patient 1 were collected at 38 and 42 years of age, corresponding to disease onset and the advanced symptomatic phase, respectively [35]. Written informed consent for tissue collection and experimental use was obtained from all participants prior to enrollment. The study was conducted in accordance with institutional ethical standards and approved by the Ethics Committee of Università Cattolica del Sacro Cuore (Protocol No. P/740/CE/2012, approved on 30 July 2012) and by the Palermo 1 Ethics Committee (Protocol No. 4/2019, approved on 29 April 2019). Fibroblasts were maintained in Dulbecco’s Modified Eagle Medium (DMEM) supplemented with 15% fetal bovine serum (FBS), 2 mM L-glutamine, 100 U/mL penicillin, and 100 μg/mL streptomycin under standard culture conditions (37 °C in a humidified atmosphere containing 5% CO_2_). HEK293T and Neuro2a cell lines were obtained from American Type Culture Collection (ATCC, Manassas, VA, USA) and cultured in DMEM supplemented with 10% FBS, 2 mM L-glutamine, and the same antibiotics. Unless otherwise specified, all cell culture reagents were purchased from Euroclone, Pero, Italy. Mycoplasma contamination was routinely assessed throughout the study using the MycoFluor™ Mycoplasma Detection Kit according to the manufacturer’s instructions. A schematic workflow of dermal fibroblast isolation, together with further details on skin biopsy collection and primary dermal fibroblast culture, is provided in the Supplementary Information (Supplementary Text S1 and Figure S1).

### 2.2. Plasmid Constructs and Transfection

The plasmids that were used in this study have been described in earlier studies [36]. The transfection of HEK293T and Neuro2a cells was carried out in accordance with the previously described method, employing Metafectene Pro (Biontex, Munich, Germany) in strict compliance with the manufacturer’s guidelines. Fibroblasts were transfected using Human Dermal Fibroblast Nucleofector Kit (VPD-1001, Lonza, Basel, Switzerland) according to the manufacturer’s instructions. Cells were processed 48 h after transfection. Transfection efficiency was assessed during preliminary optimization experiments by calculating the percentage of GFP-positive cells relative to the total number of DAPI-positive nuclei 48 h after transfection. Similar efficiencies were observed following transfection with GFP, GFP–TDP-43^WT^ and GFP–TDP-43^G376D^ within each cell line. Further details regarding plasmid construction and transfection procedures are provided in the Supplementary Information (Supplementary Text S2).

### 2.3. mtKeima Assay

Fibroblasts were transfected with pHAGE-mt-mKeima (mtKeima) (Addgene #131626) as previously described. At 24 h after transfection, cells were seeded into microscopy chambers (8-well μ-slide, Ibidi GmBh, Martinsried, Germany). The next day, they were treated with 30 µM carbonyl cyanide m-chlorophenylhydrazone (CCCP, Enzo Life Sciences, New York, NY, USA) for 2 h. mt-Keima was sequentially excited at 458 nm (neutral pH, mitochondria) and 561 nm (acidic pH, lysosomes), and emission was collected with a long-pass filter with a 605- to 695-nm emission range. For quantification, regions of interest (ROIs) corresponding to individual cells were selected using ImageJ software (version 1.5Oi, Bethesda, MD, USA). Background fluorescence was subtracted for each channel. The mitophagy index was calculated as the ratio of fluorescence intensity obtained upon excitation at 561 nm (acidic signal) to that obtained at 458 nm (neutral mitochondrial signal) for each cell (561/458 ratio).

### 2.4. TOM20/LC3B Colocalization

Fibroblasts were transfected as previously described with a plasmid encoding GFP-Microtubule Associated Protein 1, Light Chain 3 (LC3), kindly gifted by Dr. M. Chiariello (Core Research Laboratory-Siena, Institute for Cancer Research and Prevention; Institute of Clinical Physiology, National Research Council, CNR) and seeded on 11 mm round glass coverslips in 24-well plates. At 48 h after transfection, cells were treated with 30 µM CCCP for 1 h and then fixed with 4% paraformaldehyde for 15 min at room temperature, then permeabilized with 0.1% Triton X-100 in PBS for 10 min at room temperature and immunolabeled using anti-TOM20 antibody (Abcam, ab186734; Waltham, MA, USA), followed by Alexa 568-conjugated secondary antibody, both diluted in PBS-Saponin 0.1%. Coverslips were mounted using Mowiol (Calbiochem, Novabiochem Corporation, La Jolla, CA, USA). Fluorescence images were acquired using a confocal laser scanning microscope (LSM 900; Zeiss, Germany). Weighted colocalization coefficients of TOM20 and LC3 were quantified using ZEN Black Edition 2011 software (ZEN Black Edition; Zeiss, Germany).

### 2.5. Transmission Electron Microscopy

Cell monolayers were fixed in 2.5% glutaraldehyde, 2% paraformaldehyde and 2 mM calcium chloride in 0.1 M Na-cacodylate buffer (pH 7.4) overnight at 4 °C. Successively, samples were washed in buffer, post-fixed in 1% OsO4 in 0.1 M Na-cacodylate for 1 h at RT, and treated with 1% tannic acid for 30 min. Post-fixed samples were rinsed in the same buffer, dehydrated in 20% ethanol for 15 min, and en bloc stained with UA-Zero EM Stain (AGR1000; Agar Scientific, Rotherham, UK) for 1.5 h. Monolayers were then dehydrated through a graded series of ethanol solutions (20–100%) and embedded in medium-hard epoxy resin (AGR1031 Agar100 Resin Kit; Agar Scientific, Rotherham, UK). After polymerization at 65 °C for 60–72 h, ultrathin sections were obtained using a Leica EM UC6 ultramicrotome (Leica Microsystems, Wetzlar, Germany) and examined at 100 kV with a Philips EM208S transmission electron microscope (Thermo Fisher Scientific—FEI Company, Hillsboro, OR, USA), equipped with a MegaView II SIS camera (EMSIS, Milan, Italy) and RADIUS acquisition and imaging software (iTEM3/RADIUS software, version 2.1).

### 2.6. Western Blotting

For Western blot analysis, cells were detached using trypsin, collected by centrifugation, and washed with phosphate-buffered saline (PBS) prior to lysis in RIPA (radioimmunoprecipitation assay) buffer supplemented with protease inhibitor cocktail and PhosSTOP^TM^ phosphatase inhibitor cocktail (Roche Diagnostics, Mannheim, Germany), as previously described [39]. PhosSTOP^TM^ was added to prevent dephosphorylation of phosphorylation-sensitive proteins during sample preparation. Cell lysates were subjected to SDS–PAGE, and proteins were subsequently transferred onto nitrocellulose membranes (MilliporeSigma, Burlington, MA, USA). Membranes were blocked with 5% nonfat dry milk in TBS-T. Primary antibodies were diluted in TBS containing 0.01% Tween 20 and 5% Bovine Serum Albumin (BSA), whereas secondary antibodies were diluted in TBS containing 0.01% Tween 20 and 5% nonfat dry milk. After incubation with chemiluminescent substrate (Bio-Rad Laboratories, Hercules, CA, USA), immunoreactive bands were visualized using a ChemiDoc MP Imaging System (Bio-Rad Laboratories). Band intensities were quantified using Image Lab software (version 6.1; Image Lab; Bio-Rad Laboratories). For APE1 and OGG1 in Neuro2a cells, when two immunoreactive bands were detected, the intensities of both bands were measured and summed to obtain the total signal used for densitometric analysis. The antibodies used are listed in Supplementary Information (Table S1).

### 2.7. Genomic DNA Isolation and qPCR

Genomic DNA was isolated from 5 × 10^6^ HEK293T and Neuro2a cells using the Genomic-tip 20/G kit (QIAGEN, Valencia, CA, USA), according to the manufacturer’s instructions. DNA concentration and purity were assessed by spectrophotometric analysis at 260/280 nm using a NanoDrop 2000C instrument (Thermo Fisher Scientific, Waltham, MA, USA). Quantitative PCR (qPCR) was performed using 15 ng of genomic DNA as template, as previously described [40,41]. Mitochondrial DNA (mtDNA) amplification was evaluated using a long (13.4 kb) and a short (235 bp) mtDNA fragment. qPCR products were quantified using PicoGreen fluorescence dye (PicoGreen dsDNA Assay Kit), and fluorescence signals were measured with a Synergy H1 microplate reader (Agilent Technologies, Santa Clara, CA, USA). Mitochondrial DNA damage was estimated by comparing the amplification efficiency of the long fragment relative to the short fragment. Lesion frequency per 10 kb was calculated based on the Poisson distribution. Detailed methodological information is provided in the Supplementary Information (Supplementary Text S3).

### 2.8. Statistical Analysis

All statistical analyses were performed using GraphPad Prism (version 8.0.1; GraphPad Software, San Diego, CA, USA). Experiments performed in HEK293T and Neuro2a cells were independently repeated three times using cultures established from separate frozen stocks. Similarly, each patient-derived fibroblast line was analyzed in three independent experiments using cells recovered from separate frozen stocks. Data are presented as mean ± standard deviation (SD) and were confirmed to be normally distributed. Statistical comparisons among experimental groups were performed using one-way ANOVA followed by Tukey’s post hoc test. Differences were considered statistically significant at *p* ≤ 0.05, *p* ≤ 0.01, and *p* ≤ 0.001.

## 3. Results

### 3.1. TDP-43^G376D^ Induces Defective Mitophagic Flux Despite Preserved PINK1 Activation

**Figure 1:**
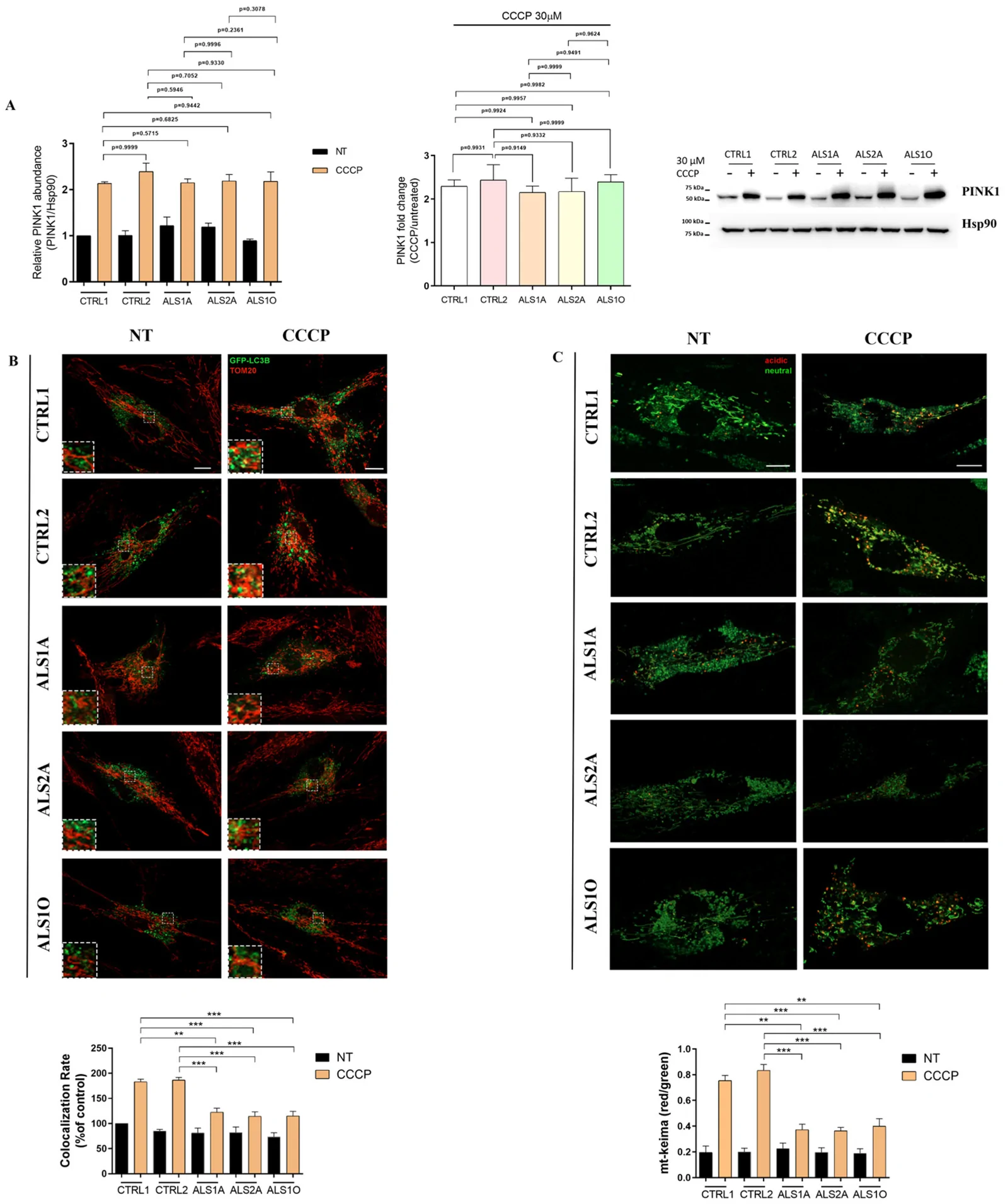
TDP-43^G376D^ affects late steps of mitophagy. (**A**) Control and ALS fibroblasts were treated with 30 μM CCCP for 1 h or maintained in complete medium and then subjected to Western blot analysis. Representative immunoblots show PINK1 and Hsp90 levels. PINK1 was normalized to Hsp90, and the CCCP-induced fold change was calculated relative to the corresponding untreated condition for each fibroblast line. Histograms show densitometric analysis from three independent experiments. In the first graph, statistical comparisons were performed within the same experimental condition (untreated or CCCP-treated). Cropping of representative images is shown in the figure. (**B**) Control and ALS fibroblasts were transfected using a plasmid encoding GFP-LC3B. At 48 h after transfection, cells were treated with CCCP and then fixed and immunolabeled with an antibody against TOM20. Scale bar = 10 µm. (**C**) Control and ALS fibroblasts were transfected with mtKeima. At 48 h after transfection, cells were treated with CCCP and live-imaged upon excitation at 561 nm (acidic signal) and 458 nm (neutral mitochondrial signal). The ratio of two signals was calculated for each cell (561/458 ratio). Scale bar = 10 µm. Data are presented as mean ± SD. Experiments using patient-derived dermal fibroblasts were independently repeated three times. Statistical significance was determined using one-way ANOVA followed by Tukey’s post hoc test for multiple comparisons. *** *p* < 0.001; ** *p* < 0.01. Abbreviations: CTRL1 and CTRL2, primary dermal fibroblasts from two healthy male donors; ALS1A and ALS2A, primary dermal fibroblasts from two male ALS patients at an advanced symptomatic stage; ALS1O, primary dermal fibroblasts from a male ALS patient at an early disease stage.

Since we previously demonstrated that lysosomal activity is impaired in TDP-43^G376D^ patient-derived cells [36], and given that mitophagy critically depends on functional lysosomes for the degradation of damaged mitochondria, we next investigated whether this pathway was affected in ALS fibroblasts. To assess the early steps of mitophagy, we first evaluated PINK1 stabilization following mitochondrial depolarization induced by CCCP treatment. As expected, CCCP exposure led to a marked accumulation of PINK1 in both control and ALS cells. No statistically significant difference in the magnitude of the CCCP-induced response was detected between the groups under the experimental conditions examined (Figure 1A).

To investigate whether autophagosome recruitment to damaged mitochondria was affected, we next analyzed the colocalization between TOM20 and LC3B following CCCP treatment. In control cells, mitochondrial depolarization induced a marked increase in LC3B–TOM20 colocalization, indicating efficient recruitment of autophagic membranes to mitochondria. In contrast, although CCCP treatment also increased LC3B–TOM20 colocalization in ALS fibroblasts, the extent of this increase was significantly lower compared to controls, suggesting reduced efficiency of mitochondrial recruitment into autophagosomes (Figure 1B).

To directly assess mitophagic flux, we performed the mtKeima assay, which allows the quantification of mitochondria delivered to acidic lysosomal compartments. Following CCCP treatment, control cells exhibited a robust increase in the red/green fluorescence ratio, consistent with efficient mitochondrial delivery to lysosomes. In contrast, ALS fibroblasts showed a significant attenuated increase in the red/green ratio compared to controls, indicating reduced mitophagic flux upon mitochondrial depolarization (Figure 1C).

### 3.2. TDP-43^G376D^ Mutation Induces Deregulation of MICOS Complex Components and Impairs Mitochondrial Cristae Organization

**Figure 2:**
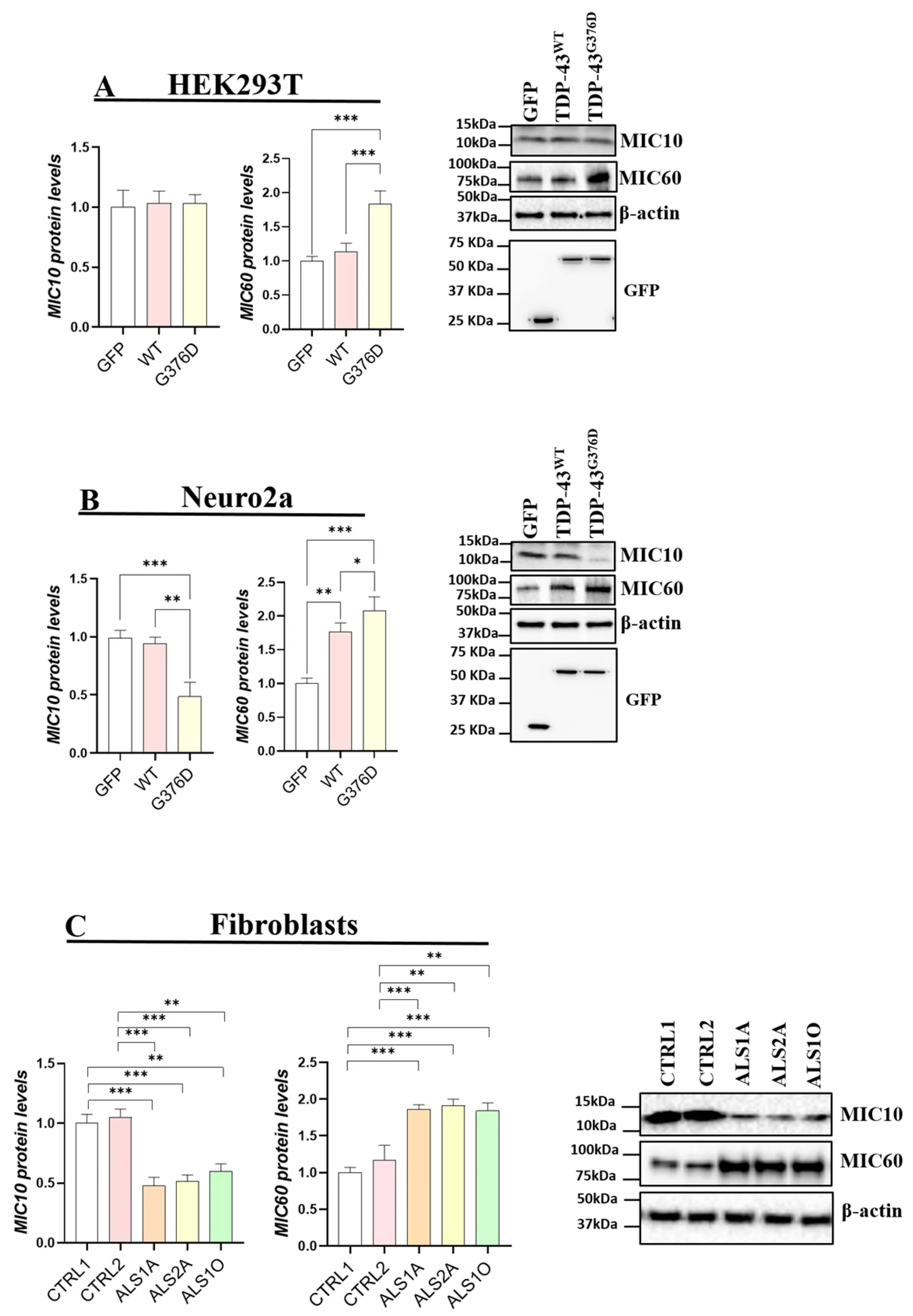
TDP-43^G376D^ Mutation impairs Mitochondrial Cristae Organization. (**A**–**C**) Representative immunoblots showing the protein expression levels of MIC10 and MIC60 in HEK293T, Neuro2a and dermal fibroblasts. Protein levels were normalized to β-actin. Representative anti-GFP Western blots confirming the expression of GFP, GFP–TDP-43^WT^ and GFP–TDP-43^G376D^ in HEK293T and Neuro2a cells 48 h after transfection are also shown. Histograms report the densitometric quantification of the immunoblots. Cropping of representative images is shown in the figure. Data are presented as mean ± SD. Experiments performed in HEK293T and Neuro2a cells were independently repeated three times. Experiments using patient-derived dermal fibroblasts were also independently repeated three times. Statistical significance was determined using one-way ANOVA followed by Tukey’s post hoc test for multiple comparisons. *p* values are indicated as follows: *** = *p* < 0.001; ** = *p* < 0.01; * = *p* < 0.05. Abbreviations: GFP, cells transfected with the pEGFPC1 vector; TDP-43WT, cells transfected with the pEGFPC1-TDP-43 wild-type construct; TDP-43^G376D^, cells transfected with the pEGFPC1-TDP-43^G376D^ mutant construct; CTRL1 and CTRL2, primary dermal fibroblasts from two healthy male donors; ALS1A and ALS2A, primary dermal fibroblasts from two male ALS patients at an advanced symptomatic stage; ALS1O, primary dermal fibroblasts from a male ALS patient at an early disease stage.

The alterations in MICOS components are associated with impaired oxidative phosphorylation and mitochondrial dysfunctions [24,25,29]. To assess whether the TDP-43^G376D^ mutation affected mitochondrial cristae organization, we investigated the protein levels of MIC10 and MIC60, two core components of the MICOS complex required for mitochondrial cristae stability, in HEK293T and Neuro2a cellular models, as well as in ALS patient-derived fibroblasts. Our results showed a significant increase in MIC60 protein levels in HEK293T cells expressing the TDP-43^G376D^ mutant protein, while MIC10 levels remained unchanged (Figure 2A). Interestingly, a significant reduction in MIC10 levels occurred in Neuro2a cells expressing the TDP-43^G376D^ mutant protein compared with wild-type TDP-43-expressing cells and GFP controls (Figure 2B). In contrast, MIC60 protein was significantly increased in both wild-type and TDP-43^G376D^ cells relative to GFP group, with a further increase occurring in cells expressing TDP-43^G376D^ compared with wild-type TDP-43 counterpart (Figure 2B). Finally, a deregulation of MICOS components was also detected in dermal fibroblasts. Specifically, MIC10 and MIC60 protein levels were significantly decreased and increased, respectively, in ALS fibroblasts compared with controls (Figure 2C), indicating a mutation-dependent alteration in cristae organization that is consistent across cell models.

**Figure 3:**
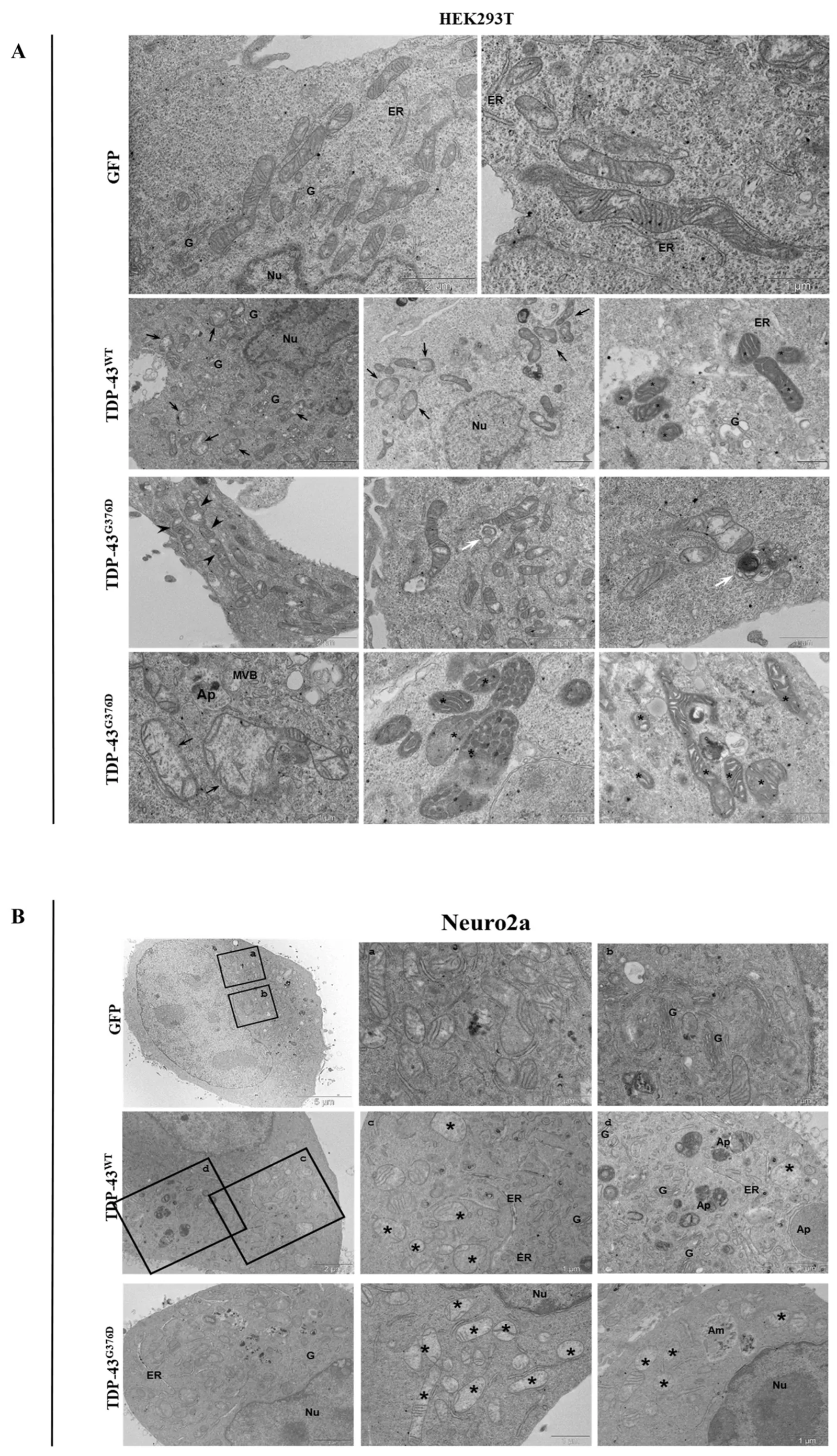
TEM analysis of HEK293T and Neuro2a cells expressing wild-type TDP-43, TDP-43^G376D^ and GFP. (**A**) The images indicate that over-expression of wild-type TDP-43 affected mitochondrial cristae organization, leading to mitochondria swelling and, in some instances, their disruption (left and middle microphotographs, arrows). In other cases, mitochondria cristae exhibited inner space dilatation accompanied by matrix condensation (right microphotograph, asterisks). In TDP-43^G376D^-expressing cells, mitochondria displayed extensive cristae rupture and re-assembly into double-membrane rings/vesicles (left microphotograph, arrowheads) or myelin-like figures (middle and right microphotographs, white arrows). Additional alterations included cristae dilatation and vesiculation of the condensed mitochondrial matrix (middle and right microphotographs, asterisks). (**B**) The over-expression of both the wild-type protein or the mutant form induced a pronounced and consistent disruption of mitochondrial cristae compared with control cells (middle column microphotophraphs, asterisks). In addition, we observed dilation and elongation of the ER cisternae throughout the cytoplasm and around organelles (right column). Golgi cisternae were also disorganized and fragmented (right column microphotophraphs). Abbreviations. ER: endoplasmic reticulum; Nu: nucleus; G: Golgi cisternae; Ap: autophagosome; Am: amphisome.

Consistent with the observed molecular features, TEM analyses revealed a significant alteration of mitochondrial cristae organization in all cellular models. Notably, wild-type TDP-43-expressing HEK293T displayed mitochondria with a reduced number of cristae and a swollen and decondensed clear matrix. In some cases, mitochondria cristae exhibited inner space dilatation accompanied by matrix condensation (Figure 3A). In the HEK293T cells expressing the TDP-43^G376D^ mutant protein, mitochondria displayed extensive cristae rupture and re-assembly into double membrane rings/vesicles or myelin-like figures (Figure 3A). Additional alterations included cristae dilatation and vesiculation of the condensed mitochondrial matrix (Figure 3A). In Neuro2a cells, both wild-type TDP-43 and TDP-43^G376D^-expressing cells displayed disruption of mitochondrial cristae, similar to that observed in HEK293T cells (Figure 3B).

**Figure 4:**
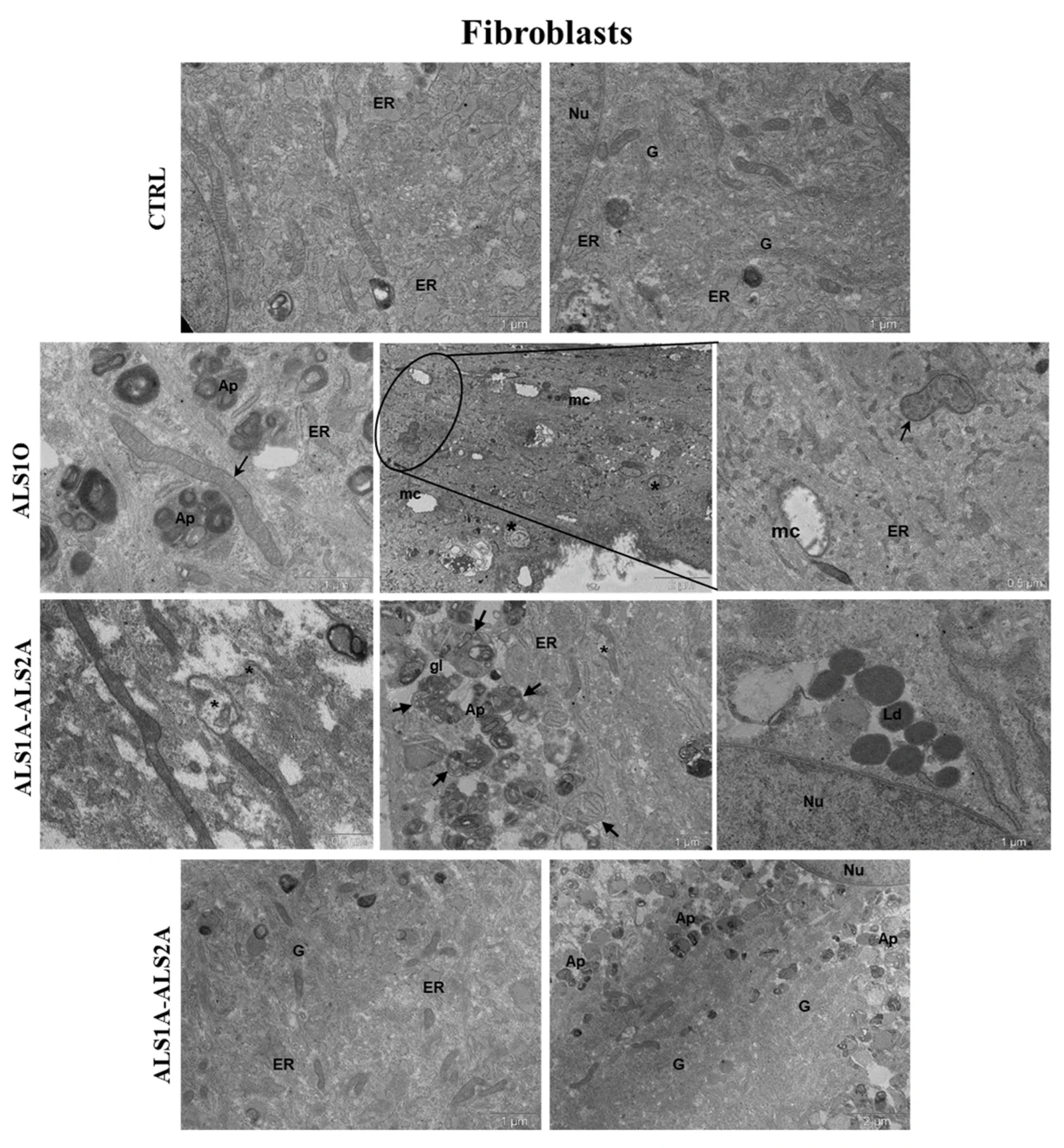
TEM analysis of control and ALS fibroblasts. In a subset of ALS1O cells, mitochondria exhibited disorganized and vesiculated cristae (left microphotoghraphs, arrow) or had partially (middle and right microphotographs asterisks) or completely (middle and right microphotographs, mc) lost their matrix. Autophagosomes were more widely distributed compared with control cells (left image, Ap). In ALS1A and ALS2A fibroblasts, mitochondrial cristae appeared convoluted within partially swollen organelles (upper left microphotograph, asterisk). In several cases, mitophagy and autophagy were observed, engulfing large regions of the cytoplasm (upper middle and right microphotographs, Ap). Accumulations of electron-transparent glycogen-like material (upper middle microphotograph, gl) and electron-dense (osmiophilic) lipid droplets (upper right microphotograph, Ld) were also present. Endoplasmic reticulum and Golgi cisternae were markedly fragmented and dispersed throughout the cytoplasm in a high proportion of cells (lower microphotographs, G and ER). Abbreviations. ER: Endoplasmic Reticulum; Nu: nucleus; G: Golgi cisternae; Ap: autophagosome; Am: amphisome.

ALS1O fibroblasts exhibited, in a subset of cells, mitochondria that had partially or completely lost their matrix or disorganized mitochondria with vesiculated cristae. ALS1A and ALS2A fibroblasts showed a more pronounced mitochondrial alteration, with convoluted cristae within partially swollen organelles (Figure 4).

### 3.3. TDP-43^G376D^ Mutation Increases Mitochondrial DNA Damage and Impairs the Base Excision Repair Pathway (BER)

**Figure 5:**
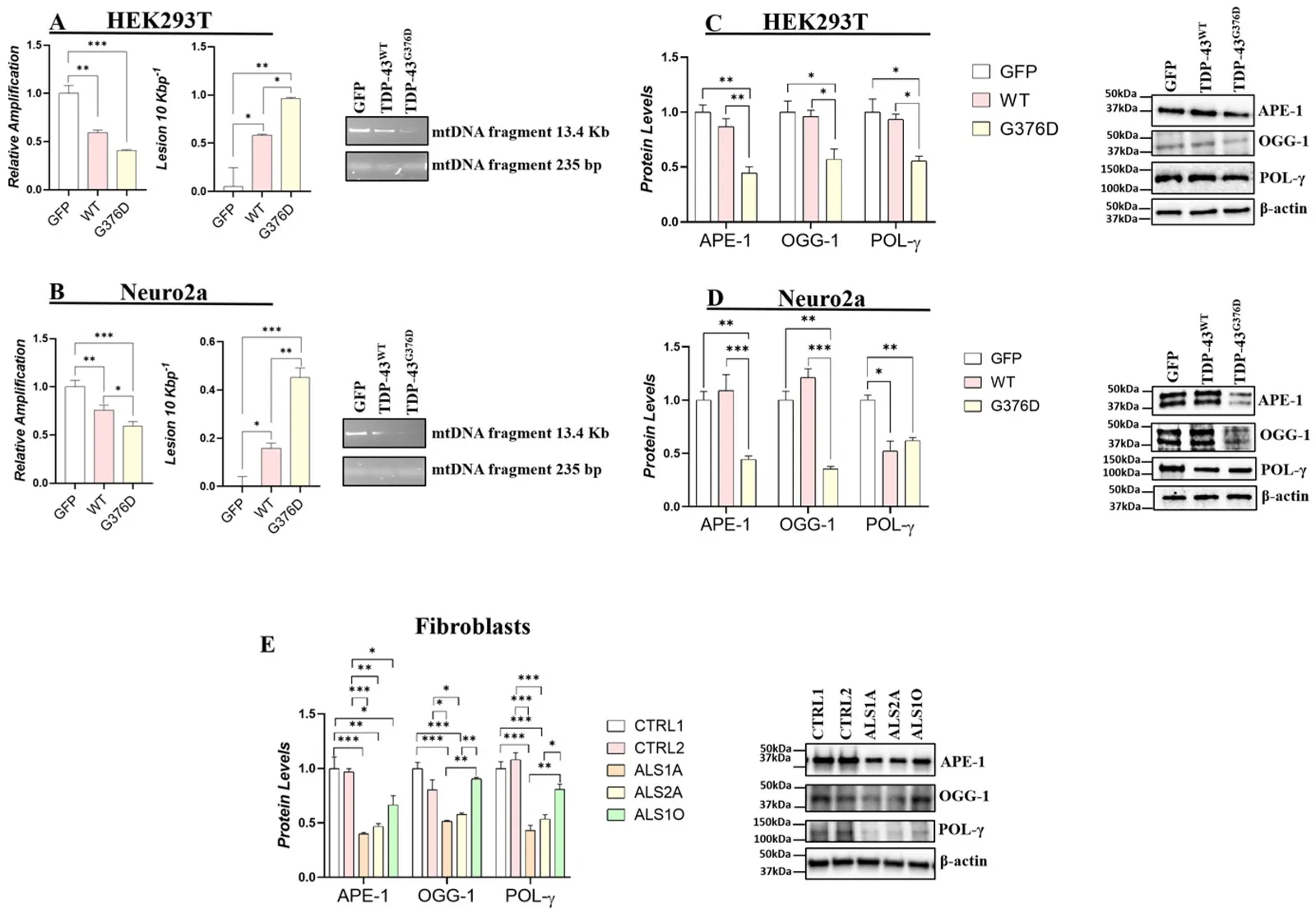
TDP-43^G376D^ Mutation Induces mtDNA Damage and BER Dysfunction. (**A**,**B**) mtDNA damage was assessed in HEK293T and Neuro2a cells by amplifying long (13.4 kb) and short (235 bp) mtDNA fragments using qPCR. Lesion frequency was calculated as the number of mtDNA lesions per 10 kb per strand. (**C**–**E**) Representative immunoblots showing the protein expression levels of APE-1, OGG-1, and POL-γ in HEK293T, Neuro2a and dermal fibroblasts. Protein levels were normalized to β-actin. Histograms report the densitometric quantification of the immunoblots. For APE1 and OGG1 in Neuro2a cells, the combined intensity of the two immunoreactive bands was used for densitometric analysis. Cropping of representative images is shown in the figure. Data are presented as mean ± SD. Experiments performed in HEK293T and Neuro2a cells were independently repeated three times. Experiments using patient-derived dermal fibroblasts were also independently repeated three times. Statistical significance was determined using one-way ANOVA followed by Tukey’s post hoc test for multiple comparisons. *p* values are indicated as follows: *** = *p* < 0.001; ** = *p* < 0.01; * = *p* < 0.05.

Given the susceptibility of mtDNA to oxidative stress, DNA damage was evaluated by quantifying lesion accumulation. A reduction in long-fragment amplification was observed in both TDP-43-expressing cell models; however, this decrease was more pronounced in cells expressing the TDP-43^G376D^ mutant compared with wild-type TDP-43 (Figure 5A–B). The relative amplification values were converted into lesion frequency using the Poisson equation. In line with these data, both cellular models exhibited significantly higher mtDNA lesion frequency compared with GFP control (Figure 5A–B). Notably, mtDNA damage was markedly greater in TDP-43^G376D^ cells than in wild-type TDP-43 counterpart. Considering the increased accumulation of mtDNA lesions, we next examined whether these alterations were associated with changes in mitochondrial DNA repair mechanisms. Key components of the BER pathway involved in mitochondrial genome maintenance, including apurinic/apyrimidinic endonuclease 1 (APE1), 8-oxoguanine DNA glycosylase (OGG1), and DNA polymerase γ, were analyzed.

Our results showed a significant reduction in APE1 and OGG1 protein levels in both HEK293T and Neuro2a cells expressing TDP-43^G376D^ compared with wild-type TDP-43 and GFP controls (Figure 5C–D). In HEK293T cells, DNA polymerase γ levels were also significantly reduced in TDP-43^G376D^-expressing cells compared with wild-type TDP-43 and GFP controls (Figure 5C). In Neuro2a cells, however, DNA polymerase γ levels were significantly reduced in both wild-type TDP-43- and TDP-43^G376D^-expressing cells compared with GFP controls (Figure 5D). In dermal fibroblasts, the protein levels of BER components were also significantly reduced in ALS cells (Figure 5E). Notably, this reduction was more pronounced in ALS1A and ALS2A than in ALS1O, indicating a progressive worsening with disease stage.

### 3.4. TDP-43^G376D^ Mutation Differentially Modulates the Canonical and Sirtuin-Dependent Axes of the UPR^mt^

**Figure 6:**
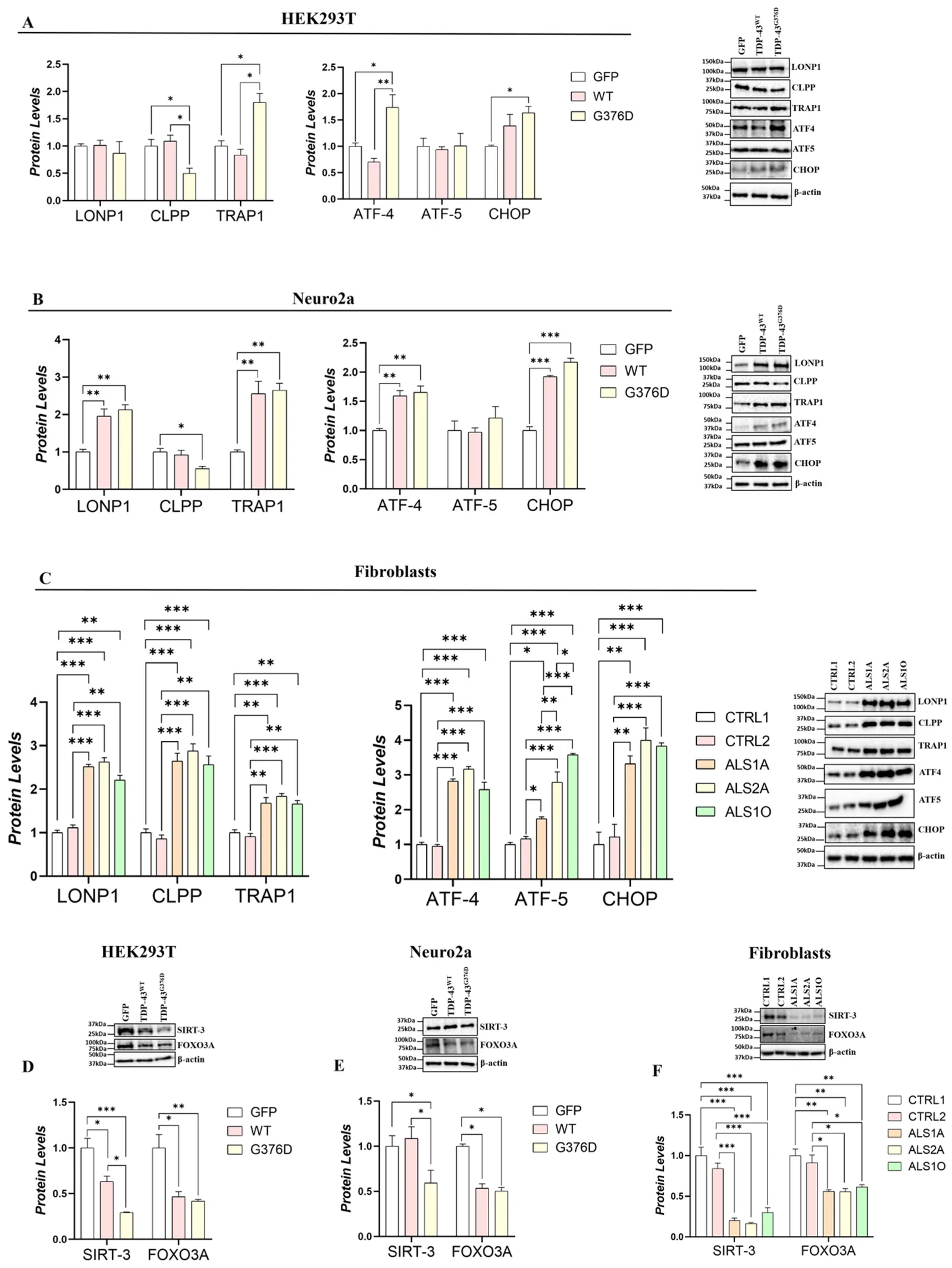
TDP-43^G376D^ differentially modulates UPR^mt^ Axes. (**A**–**C**) Representative immunoblots showing the protein expression levels of LONP1, CLPP, TRAP1, ATF-4, ATF-5 and CHOP in HEK293T, Neuro2a and dermal fibroblasts. Protein levels were normalized to B-ACTIN. Histograms report the densitometric quantification of the immunoblots. Cropping of representative images is shown in the figure. (**D**–**F**) Representative immunoblots showing the protein expression levels of SIRT-3, SIRT-7, and FOXO3A in HEK293T, Neuro2a and dermal fibroblasts. Protein levels were normalized to β-actin. Histograms report the densitometric quantification of the immunoblots. Cropping of representative images is shown in the figure. Data are presented as mean ± SD. Experiments performed in HEK293T and Neuro2a cells were independently repeated three times. Experiments using patient-derived dermal fibroblasts were also independently repeated three times. Statistical significance was determined using one-way ANOVA followed by Tukey’s post hoc test for multiple comparisons. *p* values are indicated as follows: *** = *p* < 0.001; ** = *p* < 0.01; * = *p* < 0.05.

Mitochondrial dysfunction is commonly associated with alterations in mitochondrial proteostasis and activation of stress-responsive signaling pathways, including the UPR^mt^. In HEK293T cells, protein levels of the mitochondrial protease CLPP were significantly reduced in TDP-43^G376D^-expressing cells compared with wild-type TDP-43 and GFP control (Figure 6A). In contrast, the mitochondrial chaperone TRAP1, as well as the transcription factors ATF4 and CHOP, were significantly increased in TDP-43^G376D^ cells (Figure 6A). In Neuro2a cells, LONP1, TRAP1, ATF4, and CHOP were upregulated in both wild-type and mutant TDP-43-expressing cells compared with GFP control, with a more pronounced effect in the TDP-43^G376D^ condition (Figure 6B). Similarly, ALS-derived dermal fibroblasts showed increased levels of LONP1, CLPP, TRAP1, ATF4, ATF5 and CHOP compared with healthy controls (Figure 6C). Collectively, these results indicate robust activation of the canonical UPR^mt^ axis across cellular and patient-derived models, consistent with sustained mitochondrial stress. Beyond the canonical axis, mitochondrial stress responses are also regulated by the sirtuin-dependent branch, which plays a crucial role in maintaining mitochondrial homeostasis and antioxidant defenses. Key mediators of this pathway include Sirtuin 3 (SIRT3) and Forkhead box O3a (FOXO3A). In both HEK293T and Neuro2a cells, the protein levels of these markers were significantly reduced in TDP-43^G376D^ and wild-type TDP-43-expressing cells compared with GFP controls, with a more pronounced decrease in the mutant condition (Figure 6D–E). Likewise, ALS-derived fibroblasts showed a significant reduction in SIRT3 and FOXO3A protein levels compared with healthy controls (Figure 6F). Collectively, these data indicate a selective impairment of the sirtuin-dependent UPR^mt^ branch, consistent with reduced mitochondrial antioxidant capacity.

### 3.5. TDP-43^G376D^ Mutation Induces Endoplasmic Reticulum Stress and Activates the UPR^ER^

**Figure 7:**
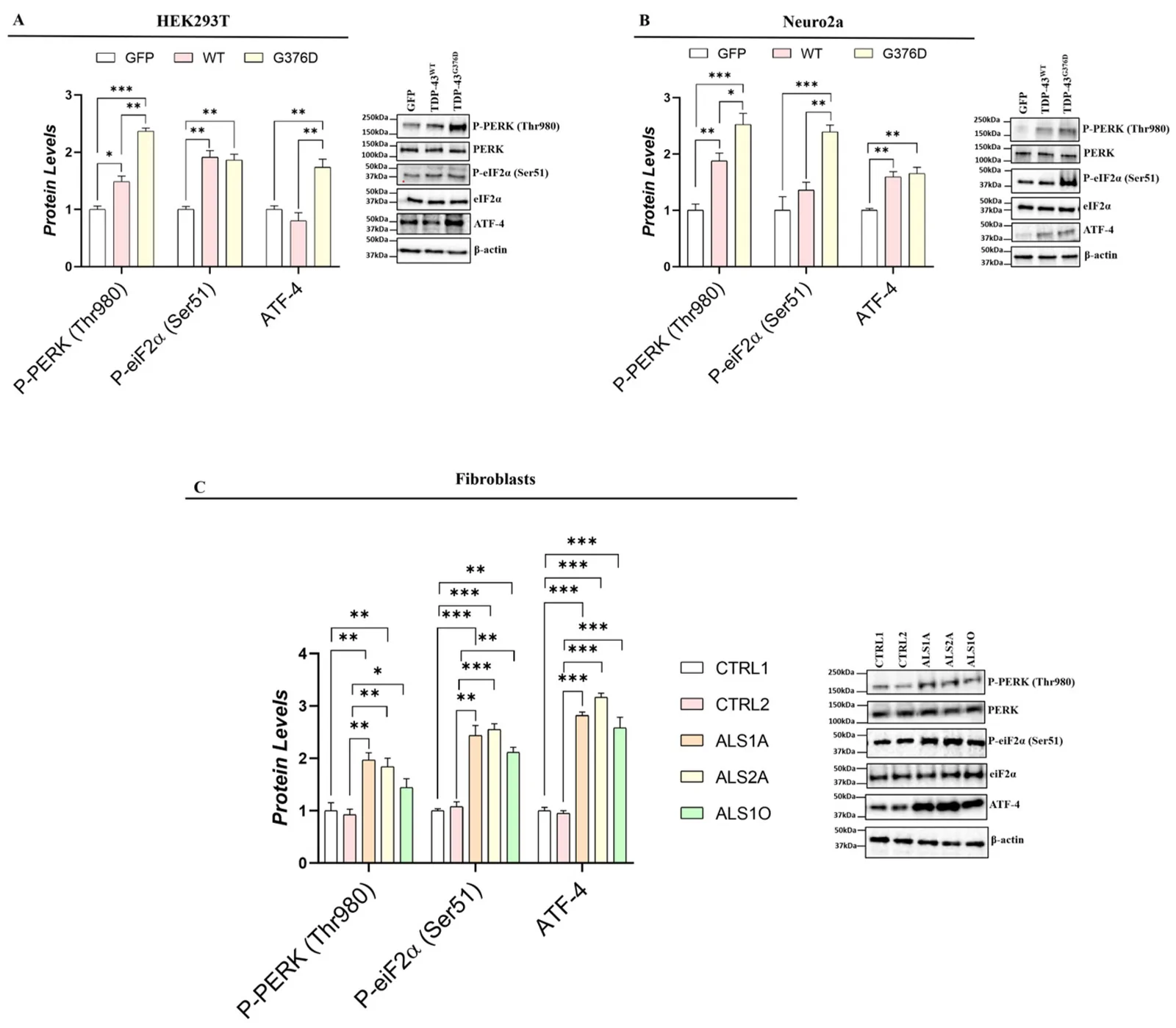
TDP-43^G376D^ Mutation Triggers Endoplasmic Reticulum Stress and Activates the UPR^ER^. (**A**–**C**) Representative immunoblots showing the protein expression levels of P-PERK (Thr980), P-eiF2α (Ser51), and ATF-4 in HEK293T, Neuro2a, and dermal fibroblasts. The ATF-4 immunoblots shown in Figure 7A–C are the same as those presented in Figure 6A–C because ATF-4 was analyzed using the same protein lysates and Western blot experiments. ATF-4 is shown in both figures because it is a shared mediator of the canonical UPR^mt^ and the PERK–eIF2α–ATF4 branch of the UPR^ER^. The protein level was normalized to that of β-actin and/or to the total forms for phosphorylated proteins. Histograms report the densitometric quantification of the immunoblots. Cropping of representative images is shown in the figure. Data are presented as mean ± SD. Experiments performed in HEK293T and Neuro2a cells were independently repeated three times. Experiments using patient-derived dermal fibroblasts were also independently repeated three times. Statistical significance was determined using one-way ANOVA followed by Tukey’s post hoc test for multiple comparisons. *p* values are indicated as follows: *** = *p* < 0.001; ** = *p* < 0.01; * = *p* < 0.05.

Chronic mitochondrial stress may extend beyond the organelle itself and disrupt the homeostasis of other intracellular compartments. Sustained oxidative stress has been shown to impair ER function, potentially leading to activation of the UPR^ER^. To assess activation of ER stress pathways, we studied the PERK–eIF2α–ATF4 signaling axis by evaluating PERK and eIF2α phosphorylation, along with ATF4 protein levels. Since ATF4 is a shared mediator of the canonical UPR^mt^ and the PERK–eIF2α–ATF4 branch of the UPR^ER^ [42,43], the same ATF4 immunoblot data are presented in Figure 6 and Figure 7 to facilitate the interpretation of its role in these interconnected stress-response pathways.

In HEK293T cells, phosphorylation of PERK (Thr980) and ATF4 protein expression were significantly increased in TDP-43^G376D^-expressing cells compared with wild-type TDP-43-expressing cells and GFP, whereas phosphorylation of eIF2α (Ser51) was significantly increased only compared with GFP (Figure 7A). In Neuro2a cells, PERK phosphorylation and ATF4 protein expression were significantly increased in both wild-type TDP-43- and TDP-43^G376D^-expressing cells compared with GFP, with PERK phosphorylation being significantly higher in TDP-43^G376D^ than in wild-type TDP-43-expressing cells. In contrast, phosphorylation of eIF2α (Ser51) was significantly increased only in TDP-43^G376D^-expressing cells compared with wild-type TDP-43 and GFP (Figure 7B).

Importantly, activation of the UPR^ER^ pathway was also detected in dermal fibroblasts derived from ALS patients carrying the mutation, which showed significantly increased PERK and eIF2α phosphorylation and elevated ATF4 protein levels compared with healthy controls (Figure 7C). Notably, this increase was more pronounced in ALS1A and ALS2A fibroblasts than in ALS1O cells, indicating progressive hyperactivation of ER stress signaling with disease progression (Figure 7C). These findings demonstrate that TDP-43^G376D^ induces a strong activation of the PERK–eIF2α–ATF4 pathway across cellular models and patient-derived fibroblasts, indicating that ER stress is a consistent feature associated with the mutation. At the ultrastructural level, dilation and elongation of ER cisternae were observed in Neuro2a cells expressing either wild-type TDP-43 or TDP-43^G376D^ (Figure 3B). Golgi cisternae also appeared disorganized and fragmented (Figure 3B). Fibroblasts from ALS1A and ALS2A patients exhibited markedly fragmented ER and Golgi cisternae distributed throughout the cytoplasm (Figure 4), affecting a high proportion of cells. Overall, these morphological features are consistent with ER stress, although this cannot be quantitatively assessed based solely on ultrastructural analysis. In contrast, no clear ultrastructural evidence of ER stress was observed in HEK293T cells, although the complexity of cytoplasmic organelles in this cell line may lead to an underestimation of such alterations.

## 4. Discussion

Mitochondrial dysfunction is increasingly recognized as a central contributor to neurodegenerative diseases, including ALS, where it underlies neuronal vulnerability and progressive cellular degeneration [44,45]. Mitochondrial homeostasis relies on a tightly coordinated network of quality control mechanisms, including proteostasis, mitophagy, mitochondrial cristae organization, and mtDNA integrity. Rather than acting as independent processes, these pathways function as an integrated network, whose coordinated disruption can amplify mitochondrial damage and cellular stress. Failure of these mechanisms results in the accumulation of damaged mitochondria, increased ROS production and reduced bioenergetic capacity, all contributing to cellular stress and cell death [46,47]. Importantly, our previous work showed that the ALS-linked TDP-43^G376D^ mutation disrupts key processes of MQC, including decreased mitochondrial biogenesis, enhanced fission, and impaired fusion, suggesting that this mutation may directly compromise mitochondrial homeostasis [35].

Based on these findings, the present study aimed to investigate additional, interconnected processes of MQC that could contribute to mitochondrial dysfunction in ALS. Specifically, we explored the impact of TDP-43^G376D^ on mitophagy, mitochondrial cristae architecture, and mtDNA integrity. Furthermore, we examined the activation of the UPR^mt^ and its crosstalk with ER stress pathways to explore how maladaptive inter-organelle stress signaling may exacerbate cellular dysfunction in ALS. Importantly, while our previous studies documented mitochondrial and lysosomal dysfunction associated with TDP-43^G376D^, the present study extends these observations by examining additional, interconnected MQC pathways. This broader characterization provides additional insights into the mitochondrial alterations associated with the TDP-43^G376D^ variant.

Mitophagy represents a key component of the MQC, and its malfunction leads to the accumulation of dysfunctional mitochondria, thereby enhancing cellular stress [48]. In ALS cells, we did not detect evidence of an altered CCCP-induced PINK1 response under the experimental conditions examined, suggesting that this early step of the mitophagy pathway may be preserved. On the contrary, the late stages of mitophagy are affected, as indicated by the reduced LC3 recruitment and the decrease in mtKeima signal. Importantly, we previously demonstrated that TDP-43^G376D^ induced lysosomal dysfunction [36]. Considering that lysosomes are required for the final degradation of mitochondria, these findings suggest that the impaired mitophagy observed here is likely due to impaired lysosomal activity rather than defective initiation of the mitophagy process. In our previous work, we demonstrated that TDP-43^G376D^ is associated with reduced mitochondrial biogenesis [35]. Interestingly, when lysosomal dysfunction represents the primary defect, mitochondrial biogenesis is reduced, likely as a compensatory mechanism to limit the burden on the autophagic system. While initially protective, this response may establish a feed-forward loop that further exacerbates both mitochondrial and lysosomal dysfunction [49]. In this context, our findings are consistent with a disruption of this balance, where lysosomal impairment and defective mitophagic flux synergistically contribute to MQC failure in TDP-43^G376D^ cells.

Defective elimination of damaged mitochondria promotes the accumulation of structurally compromised organelles, particularly affecting cristae organization. Cristae architecture is critical for efficient oxidative phosphorylation and ATP production, as it maximizes the surface area available for Electron Transport Chain (ETC) complexes [50]. As a consequence, alterations in cristae morphology can compromise bioenergetic efficiency, increase ROS production, and trigger cellular stress responses [51,52]. Consistent with defective clearance of damaged mitochondria, structural remodeling of the mitochondrial cristae emerges as an additional layer of dysfunction. Our results show that the TDP-43^G376D^ mutation induces pronounced alterations in mitochondrial cristae organization in HEK293T, Neuro2a, and ALS patient-derived fibroblasts. Molecular analyses also revealed a mutation-dependent dysregulation of MICOS components, with changes in MIC10 and MIC60 protein levels, suggesting impaired maintenance of cristae structure. Consistent with these molecular features, TEM analyses confirmed severe disruption of mitochondrial cristae in both wild-type TDP-43 and TDP-43^G376D^-expressing cells, with more pronounced alterations in the mutant condition. These alterations included reduced cristae number, matrix swelling and decondensation, and cristae lumen dilation with matrix condensation. In TDP-43^G376D^-expressing cells, extensive cristae rupture and reassembly into double-membrane vesicles were also observed.

In addition, ALS patient-derived fibroblasts carrying the TDP-43^G376D^ mutation displayed mitochondria with partial or complete loss of matrix and disorganized, vesiculated cristae, particularly in advanced disease stages, where mitochondria showed highly convoluted cristae within swollen organelles. These structural defects are consistent with previous observations of profound mitochondrial bioenergetic impairments, including reduced ATP production, decreased membrane potential, and increased ROS generation [35,53,54].

Defective mitophagic flux may contribute to the accumulation of damaged mitochondria, thereby increasing the susceptibility of mtDNA to oxidative lesions [55,56,57]. Concomitantly, alterations in mitochondrial cristae architecture compromise bioenergetic efficiency and elevate ROS production, further exacerbating mtDNA damage [50,56,58]. These mitochondrial dysfunctions collectively promote the progressive accumulation of oxidative DNA damage in neurons, and particularly mtDNA, which is thought to play a critical role in ALS pathogenesis [59,60,61].

Consistent with this, our results demonstrate a significant increase in mtDNA damage in both HEK293T and Neuro2a cells expressing TDP-43, with the effect being markedly more pronounced in cells carrying the TDP-43^G376D^ mutation. Similarly, increased mtDNA damage has been reported in fibroblasts derived from ALS patients harboring the TDP-43^M337V^ mutation compared to controls [62,63]. Importantly, the observed increase in mtDNA lesions was accompanied by a reduction in key components of the BER pathway, suggesting that impaired repair mechanisms may further exacerbate the accumulation of oxidative mtDNA damage. Taken together, these findings indicate that TDP-43^G376D^ not only promotes mtDNA damage but also impairs the mitochondrial DNA repair machinery, thereby establishing a vicious cycle that further aggravates mitochondrial dysfunction.

Our previously reported findings, which highlighted impaired mitochondrial bioenergetics, reduced ATP production, and increased ROS generation [35], together with the current results showing TDP-43^G376D^-induced mitochondrial cristae disruption, altered mitophagy, and compromised mtDNA repair, underscore a profound mitochondrial dysfunction. These findings collectively indicate disruption of multiple interconnected MQC pathways. These observations prompted us to investigate whether the TDP-43^G376D^ mutation activates the canonical axis of UPR^mt^, a pathway whose overactivation has been reported to produce detrimental effects and contribute to neurodegenerative diseases, including the worsening of disease symptoms in an ALS mouse model [15,64]. Protein levels of mitochondrial chaperones and proteases, as well as the transcription factors ATF4 and CHOP, were markedly increased in TDP-43^G376D^-expressing HEK293T and Neuro2a cells, as well as in ALS patient-derived fibroblasts, reflecting a sustained mitochondrial stress response. This persistent activation suggests a compensatory attempt to restore mitochondrial proteostasis that may become maladaptive under chronic stress [10,29,65].

Interestingly, while the canonical axis was engaged, the sirtuin-dependent branch of the UPR^mt^—including SIRT-3 and the downstream effector FOXO3A—was selectively impaired in cells carrying the TDP-43^G376D^ mutation. This imbalance between the adaptive and protective branches of the UPR^mt^ suggests a dysregulated stress response rather than a fully effective compensatory mechanism. Consistently, previous observations [35] showed that TDP-43^G376D^-expressing cells exhibited reduced mitochondrial antioxidant defenses alongside increased ROS production, reinforcing the notion of altered mitochondrial function.

Moreover, mitochondrial stress is known to influence ER function, and conversely, ER stress can impact mitochondrial homeostasis, creating a maladaptive inter-organelle feedback loop [65,66]. In neurodegenerative diseases, including ALS, persistent ER stress contributes to neuronal dysfunction and can further exacerbate mitochondrial alteration [67,68,69]. Our data show that chronic mitochondrial stress induced by the ALS-linked TDP-43^G376D^ mutation is accompanied by activation of the PERK–eIF2α–ATF4 branch of the UPR^ER^, supporting the notion that mitochondrial and ER stress responses are tightly interconnected in the context of ALS pathology.

This condition was consistently observed across both cellular models (HEK293T and Neuro2a) and patient-derived fibroblasts, with the most pronounced activation seen in fibroblasts from advanced-stage disease. Ultrastructural analyses further corroborated these findings, revealing dilated and fragmented ER and Golgi cisternae, consistent with a sustained ER stress response. Similar results have been reported in ALS models carrying other TDP-43, C9ORF72, or SOD1 mutations, suggesting that the ER stress response is a convergent feature of ALS pathology [67,70,71]. This study has some limitations that should be acknowledged. First, although patient-derived dermal fibroblasts represent a valuable human model to investigate disease-associated mitochondrial alterations [72], they do not fully recapitulate the tissue-specific properties and selective vulnerability of motor neurons or skeletal muscle. Due to the rarity of the TDP-43^G376D^ mutation and the limited availability of biological specimens from affected individuals, we were unable to validate our findings in disease-relevant tissues, such as skeletal muscle or central nervous system samples from mutation carriers. Moreover, the number of independent donors was limited, and ALS1O and ALS1A represent longitudinal samples obtained from the same patient and therefore cannot be considered independent biological donors. These factors limit the generalizability of our findings to unrelated p.G376D carriers, other *TARDBP* variants and sporadic ALS. Furthermore, the absence of isogenic control lines prevents the observed alterations from being attributed exclusively to the p.G376D variant, and the possible contribution of other genetic modifiers cannot be formally excluded. Future studies involving additional patient-derived samples, isogenic models, patient-derived motor neurons or neuromuscular systems will be important to validate and extend our findings.

## 5. Conclusions

In conclusion, our study identifies alterations in multiple MQC pathways associated with the TDP-43^G376D^ variant. Indeed, the late stages of mitophagy are impaired, likely as a consequence of lysosomal dysfunction. This defective clearance is associated with alterations in mitochondrial cristae architecture, increased mtDNA damage, and impairment of mitochondrial DNA repair mechanisms. Moreover, mutant TDP-43 is associated with sustained activation of the canonical UPR^mt^ axis while negatively affecting the sirtuin-dependent antioxidant axis, suggesting a maladaptive stress response. Mitochondrial dysfunction is also accompanied by activation of the ER stress pathway, highlighting the existence of interconnected mitochondrial and ER stress responses.

Our findings support a model in which TDP-43^G376D^ is associated with alterations in multiple interconnected MQC processes, accompanied by the accumulation of structurally and functionally compromised mitochondria and increased cellular stress in the cellular models examined. These findings extend our previous observations of TDP-43^G376D^-associated mitochondrial and lysosomal dysfunction by providing evidence for broader alterations across interconnected MQC pathways. Further studies are necessary to fully elucidate the molecular mechanisms through which mutant TDP-43 is linked to MQC dysfunction, and it will also be important to determine whether these alterations arise from a primary effect of TDP-43 on mitochondrial homeostasis or from its cytoplasmic aggregation and mislocalization. Furthermore, it will be necessary to validate these findings in neuronal models and to assess their therapeutic relevance, in order to determine whether targeting MQC network dysfunction may represent a viable strategy to restore mitochondrial homeostasis in ALS.

## Data Availability

All data generated and analyzed during this study are provided within this published article and its Supplementary Information Files. Further inquiries can be directed to the corresponding authors.

## Supplementary Materials

The following supporting information can be downloaded at: https://www.mdpi.com/article/10.3390/antiox15091051/s1, Supplementary Information includes detailed methods for collection of skin biopsies and isolation of patient-derived dermal fibroblasts, plasmid constructs, transfection procedures, genomic DNA isolation and qPCR analysis (Texts S1–S3). Supplementary Table S1 lists the antibodies used for Western blot analysis. Supplementary Figure S1 shows a schematic workflow of skin biopsy collection and dermal fibroblast isolation. Supplementary Figures S2–S11 show uncropped Western blot images.

## Abbreviations

ALS	Amyotrophic Lateral Sclerosis
APE1	Apurinic/Apyrimidinic Endonuclease 1
ATF4	Activating Transcription Factor 4
ATF5	Activating Transcription Factor 5
BER	Base Excision Repair pathway
C9ORF72	Chromosome 9 Open Reading Frame 72
CCCP	Carbonyl Cyanide m-Chlorophenylhydrazone
CHOP	C/EBP Homologous Protein (DDIT3)
CLPP	Caseinolytic mitochondrial matrix peptidase proteolytic subunit
DMEM	Dulbecco’s Modified Eagle Medium
DNA polymerase γ	Mitochondrial DNA polymerase gamma
eIF2α	Eukaryotic Initiation Factor 2 alpha
ER	Endoplasmic Reticulum
ETC	Electron Transport Chain
FBS	Fetal Bovine Serum
FOXO3A	Forkhead Box O3
GFP	Green Fluorescent Protein
LC3	Microtubule-associated protein 1 light chain 3
LONP1	Lon Peptidase 1 Mitochondrial
MIC10	MICOS Complex Subunit MIC10 (C1ORF151)
MIC60	MICOS Complex Subunit MIC60 (IMMT)
MICOS	Mitochondrial Contact Site and Cristae Organizing System
MQC	Mitochondrial Quality Control
mtDNA	Mitochondrial DNA
OGG1	8-Oxoguanine DNA Glycosylase 1
PBS	Phosphate-Buffered Saline
PERK	Protein kinase R (PKR)-like endoplasmic reticulum kinase
PINK1	PTEN-induced kinase 1
qPCR	Quantitative Polymerase Chain Reaction
RIPA	Radioimmunoprecipitation Assay buffer
ROIs	Regions of Interest
ROS	Reactive Oxygen Species
SDS-PAGE	Sodium Dodecyl Sulfate–Polyacrylamide Gel Electrophoresis
SIRT3	Sirtuin 3
SOD1	Superoxide Dismutase 1.
TARDBP	TAR DNA Binding Protein gene
TBS-T	Tris-Buffered Saline with Tween 20
TDP-43	TAR DNA-binding protein 43
TEM	Transmission Electron Microscopy
TOM20	Translocase of Outer Mitochondrial Membrane 20
TRAP1	TNF Receptor Associated Protein 1
UPR^ER^	Endoplasmic Reticulum Unfolded Protein Response
UPR^mt^	Mitochondrial Unfolded Protein Response
