# Supplementary material for: Mitochondrial Quality Control Impairment Is a Hallmark of TDP-43G376D ALS Patient-Derived Fibroblasts
Source: Antioxidants (Basel). 2026 Aug 22;15(9):1051. doi: 10.3390/antiox15091051 (PMC13603339; doi:10.3390/antiox15091051)
Supplement: Supplementary file 1 [file antioxidants-15-01051-s001.zip › antioxidants-4447485-supplementary.PDF]

Mitochondrial Quality Control impairment is a hallmark of TDP-43^{G376D} ALS patient-derived fibroblasts

Giuseppe Petito[1], Maria Ventriglia[1], Victoria Stefania Del Fiore[2], Arianna Cuomo[1], Federica Cioffi[3], Francesco Manfrevola[4], Flora Guerra[5], Lucia Bertuccini[6], Giulia Ricci[4], Gilda Cobellis[4], Antonia Lanni[1], Cecilia Bucci[2], Roberta Romano[2*] and Rosalba Senese[1*]

[1]Department of Environmental, Biological and Pharmaceutical Sciences and Technologies, University of Campania "L. Vanvitelli", 81100 Caserta, Italy; giuseppe.petito@unicampania.it (G.P); ma-ria.ventriglia@unicampania.it (M.V); arianna.cuomo@unicampania.it (A.C); antonia.lanni@unicampania.it (A.L); rosalba.senese@unicampania.it (R.S)

[2]Department of Experimental Medicine (DiMeS), University of Salento, Via Provinciale Lecce-Monteroni n.165, 73100 Lecce, Italy; victoriastefania.delfiore@unisalento.it (V.S.D.F); cecilia.bucci@unisalento.it (C.B); rob-erta.romano@unisalento.it (R.R)

[3]Department of Sciences and Technologies, University of Sannio, 82100 Benevento, Italy; federi-ca.cioffi@unisannio.it (F.C)

[4]Department of Experimental Medicine, University of Campania "L. Vanvitelli", 80138 Naples, Italy; frances-co.manfrevola@unicampania.it (F.M); giulia.ricci@unicampania.it (G.R); gilda.cobellis@unicampania.it (G.C)

[5]Department of Biological and Environmental Sciences and Technologies (DiSTeBA), University of Salento, Via Provinciale Lecce-Monteroni n.165, 73100 Lecce, Italy; flora.guerra@unisalento.it (F.G)

[6]Core Facilities Microscopy Area, Italian National Institute of Health, Viale Regina Elena n. 299, 00161 Rome, Italy; lucia.bertuccini@iss.it (L.B)

*Corresponding author: Rosalba Senese, Via Vivaldi 43, 81100 Caserta, Italy (Tel: +39 0823274580; email: rosalba.senese@unicampania.it)
Roberta Romano, Via Provinciale Lecce-Monteroni 165, 73100 Lecce, Italy (roberta.romano@unisalento.it)

Supplementary Text

S1. Collection of Skin Biopsies and Isolation of Patient-Derived Dermal Fibroblasts

Skin biopsies were obtained using a sterile disposable punch biopsy instrument (4–5 mm in diameter) and immediately transferred into a sterile solution. For fibroblast isolation, tissue specimens were mechanically minced into small fragments and seeded onto 35 mm culture dishes preconditioned with Fetal Bovine Serum (FBS), followed by incubation overnight at 37°C in a humidified atmosphere containing 5% CO_2.

On the following day, tissue explants were maintained in high-glucose Dulbecco's Modified Eagle Medium (DMEM) supplemented with 15% FBS, 2 mM L-glutamine, 100 U/mL penicillin, 100 μg/mL streptomycin, and 1% non-essential amino acids. Explants were cultured under standard conditions for primary dermal fibroblasts until cell growth from the tissue fragments was observed. Once established, fibroblasts were expanded in 100 mm culture dishes and passaged upon reaching approximately 80% confluence.

S.2 Plasmid Constructs and Transfection

Full-length TDP-43 cDNA was used as template for PCR amplification of the complete TDP-43 coding sequence, which was subsequently subcloned into the pEGFP-C1 vector to generate the N-terminal GFP-tagged full-length TDP-43 construct (GFP–TDP-43). The GFP–TDP-43^{G376D} construct was generated by site-directed mutagenesis starting from the full-length GFP–TDP-43 plasmid using PfuTurbo DNA Polymerase (600252-52, Agilent Technologies, Santa Clara, CA, USA), according to the manufacturer's instructions.

Mutagenesis was performed using the following primers:

5′-GGAAATAACTCTTATATAGTGACTCTAATTCTGGTGCAGC-3′

5′-GCTGCACCAGAATTAGAGTCACTATAAGAGTTATTTCC-3′.

The empty pEGFP-C1 vector was used as control in transfection experiments.

S.3 Genomic DNA isolation and qPCR

Genomic DNA was isolated from 5×10^6 HEK293T and Neuro2a cells using the Genomic-tip 20/G kit (QIAGEN, Valencia, CA, USA), according to the manufacturer's instructions. DNA concentration and purity were assessed by spectrophotometric measurement at 260/280 nm using a NanoDrop 2000C (Waltham, MA, USA). mtDNA integrity was evaluated by amplification of a long (13.4 kb) fragment and a short (235 bp) fragment, the latter used as an internal control.

Long fragment (13.4 kb): 5'-AAAATCCCCGCAAACAATGACCACCC-3' (forward) and 5'-GGCAATTAAGAGTGGGATGGAGCCAA-3' (reverse) (accession number NC_005089.1)
Short fragment (235 bp): 5'-CCTCCCATTCATTATCGCCGCCCTGC-3' (forward) and 5'-GTCTGGGTCTCCTAGTAGGTCTGGGAA-3' (reverse) (accession number NC_005089.1)

qPCR products were quantified using PicoGreen dsDNA fluorescence assay (PicoGreen dsDNA Assay Kit), and fluorescence intensity was measured using a fluorescence microplate reader BioTek Synergy H1 (Agilent Technologies, Santa Clara, CA, USA). Mitochondrial DNA damage was quantified by comparing the relative amplification efficiency of the long mtDNA fragment normalized to the short fragment within the same sample. Specifically, relative lesion frequencies per 10 Kbp DNA was calculated by applying the Poisson distribution according to the formula:

$$Lesion\ frequency = -\ lnAd\ /Ao$$

where Ad means amplification of treated samples, and Ao represents amplification of controls.

Supplementary Table

Table S1. Antibodies used for Western blot

Antibody	Reference number
APE1	cod.NB100-116 (Novus Biologicals, LLC, Centennial, CO, USA)
ATF4	cod.#A18687 (ABclonal Technology, Woburn, MA, USA)
ATF5	cod.#A3563 (ABclonal Technology, Woburn, MA, USA)
B-ACTIN	cod.bs-0061R (Bioss Antibodies, Woburn, MA, USA)
Hsp90	sc-13119 (Santa Cruz Biotechnology, Dallas, TX, USA)
CHOP	cod.#A0221 (ABclonal Technology, Woburn, MA, USA)
CLPP	cod.#A32214 (ABclonal Technology, Woburn, MA, USA)
DNA pol γ	cod.sc-5931 (Santa Cruz Biotechnology, Dallas, TX, USA)
eiF2α (Ser51)	cod.#A0764 (ABclonal Technology, Woburn, MA, USA)
FOXO3A	cod.#A0102 (ABclonal Technology, Woburn, MA, USA)
GFP	sc-9996 (Santa Cruz Biotechnology, Dallas, TX, USA)
LONP1	cod.#A4293 (ABclonal Technology, Woburn, MA, USA)
MICOS Complex Subunit MIC10 (C1ORF151)	cod.PA5-116347 (Invitrogen, Thermo Fisher Scientific, Waltham, MA, USA)
MICOS Complex Subunit MIC60 (IMMT)	cod.abx235199 (Abbexa, Cambridge, UK)
OGG1	cod.#NB100-106 (Novus Biologicals, LLC, Centennial, CO, USA)
P-eiF2α (Ser51)	cod.#3398 (Cell Signaling Technology, Danvers, MA, USA)
PERK	cod.#3192 (Cell Signaling Technology, Danvers, MA, USA)
PINK1	cod.#6946 (Cell Signaling Technology, Danvers, MA, USA)
P-PERK (Thr980)	cod.#3179 (Cell Signaling Technology, Danvers, MA, USA)

SIRT3	cod.#2627 (Cell Signaling Technology, Danvers, MA, USA)
TRAP1	cod.#A2748 (ABclonal Technology, Woburn, MA, USA)
Anti-rabbit IgG	cod.#ab97051 (Abcam, Cambridge, UK)
Anti-mouse IgG	cod.#ab97023 (Abcam, Cambridge, UK)

Supplementary Figures

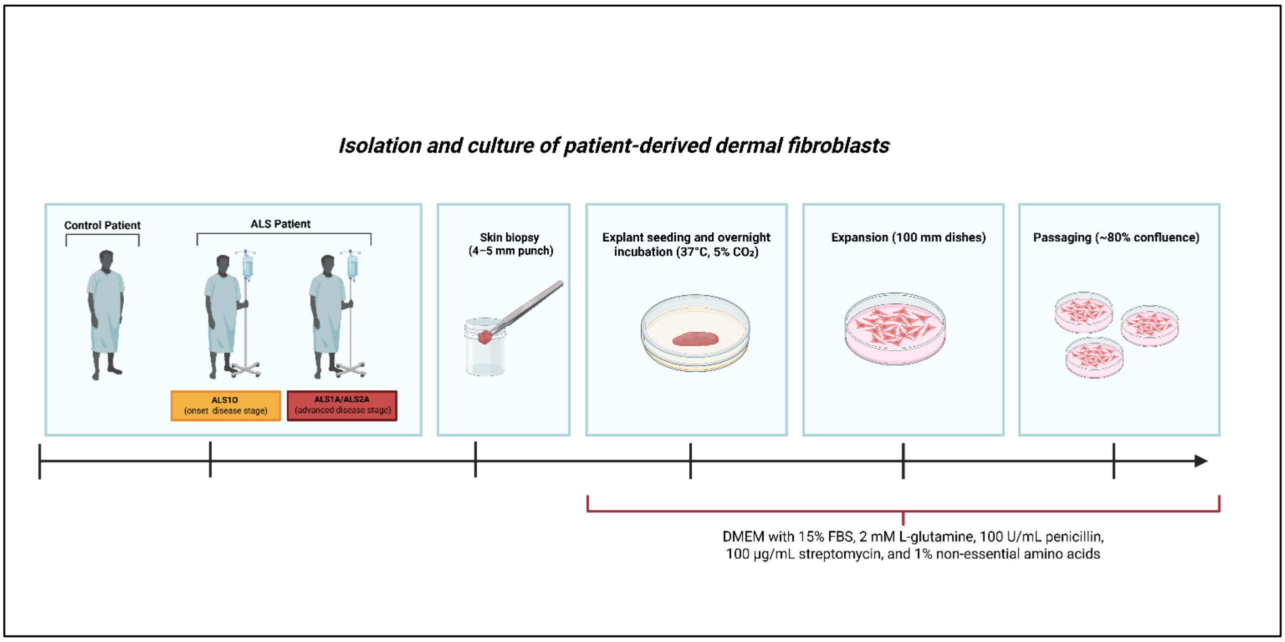

Figure S1. Schematic workflow of skin biopsy collection and dermal fibroblast isolation. ALS-derived fibroblasts were designated as ALS1A and ALS2A, corresponding to advanced symptomatic stages (A = advanced), and ALS1O, corresponding to the disease onset stage (O = onset).

Figure 1A

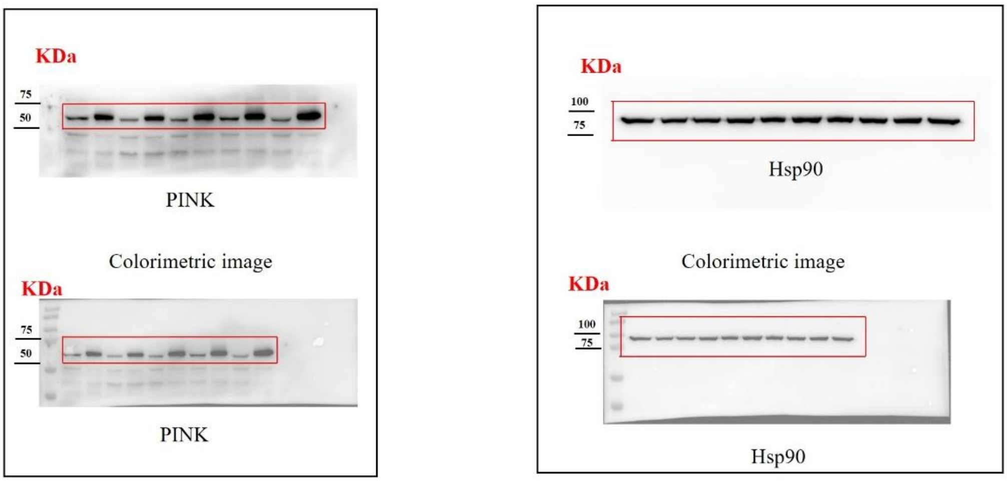

Figure 2A

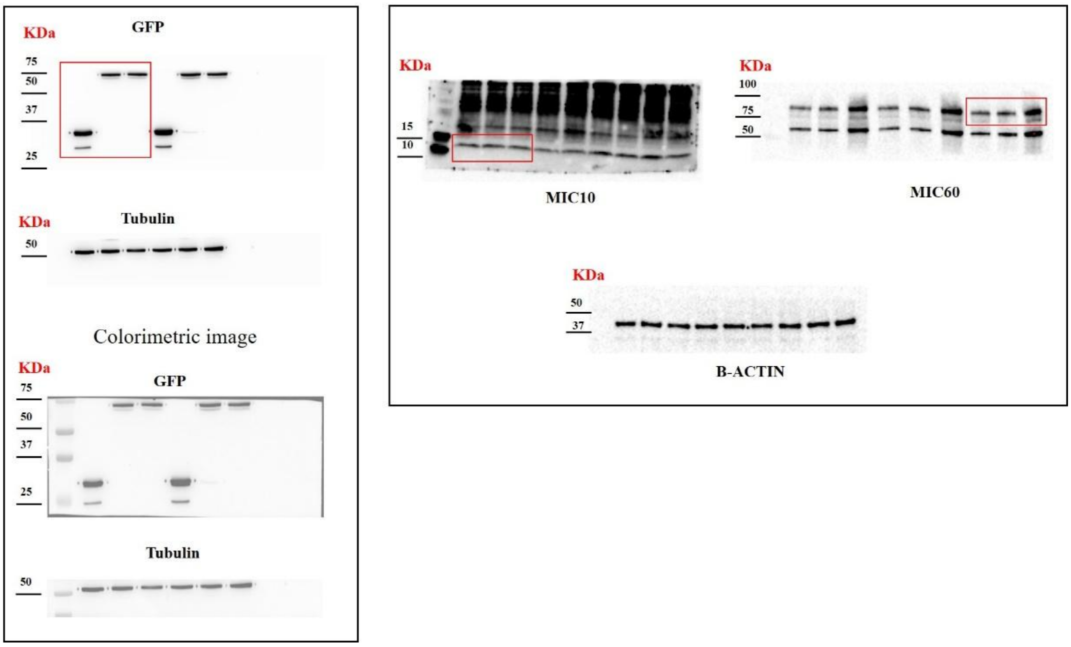

Figure S2. Uncropped Western blot images

Figure 2B

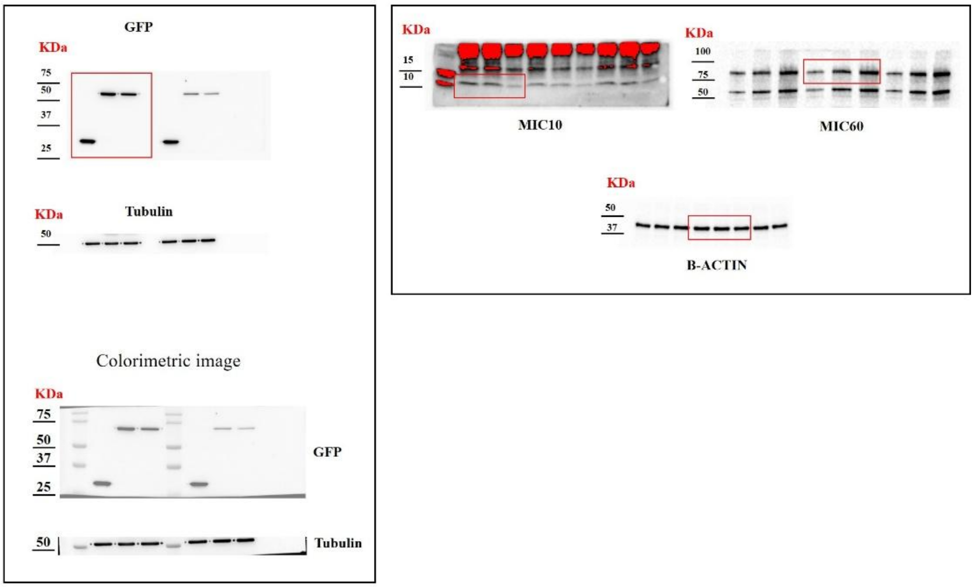

Figure 2C

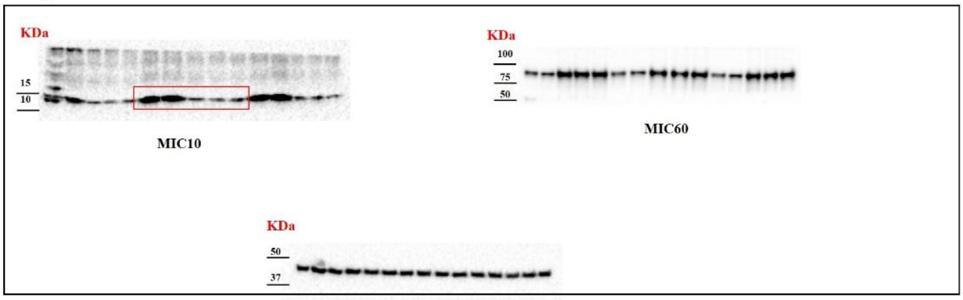

Figure S3. Uncropped Western blot images

Figure 5C

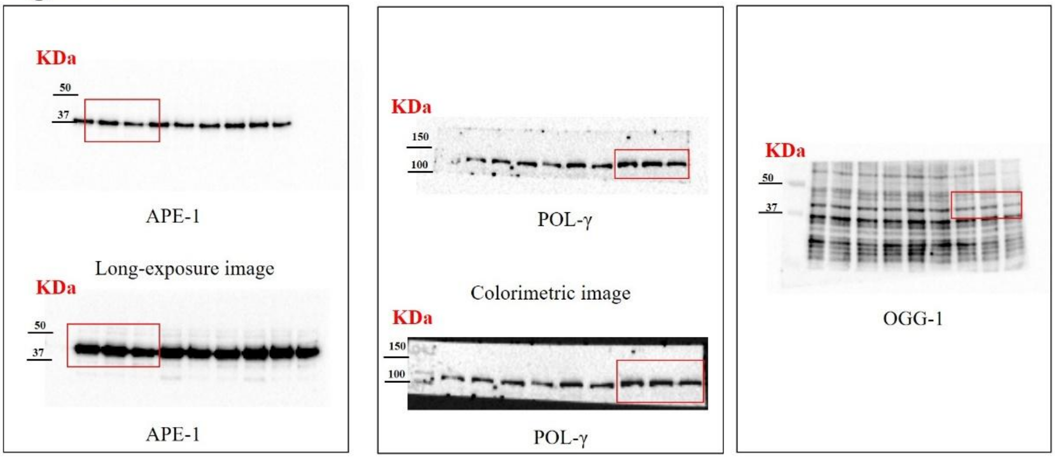

Figure 5D

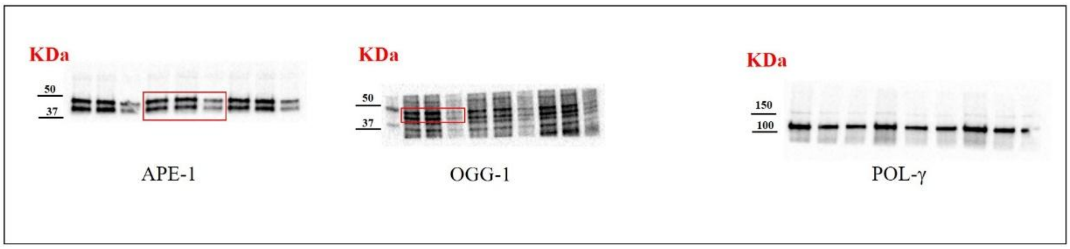

Figure 5E

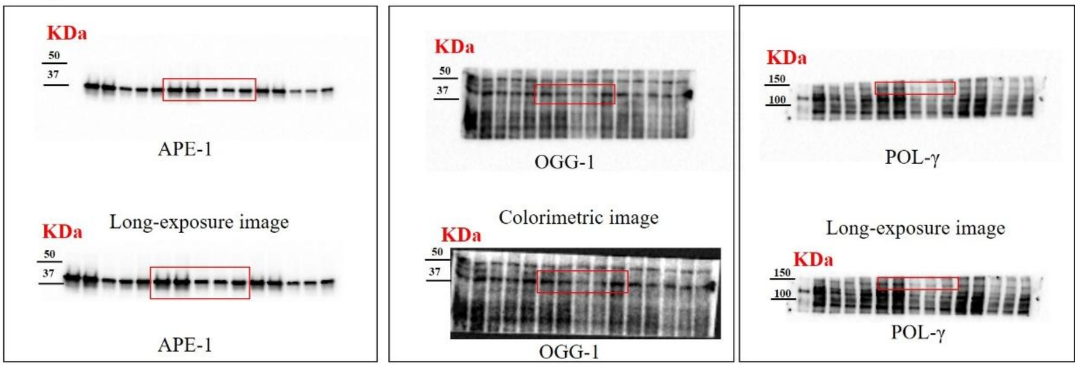

Figure S4. Uncropped Western blot images

Figure 6A

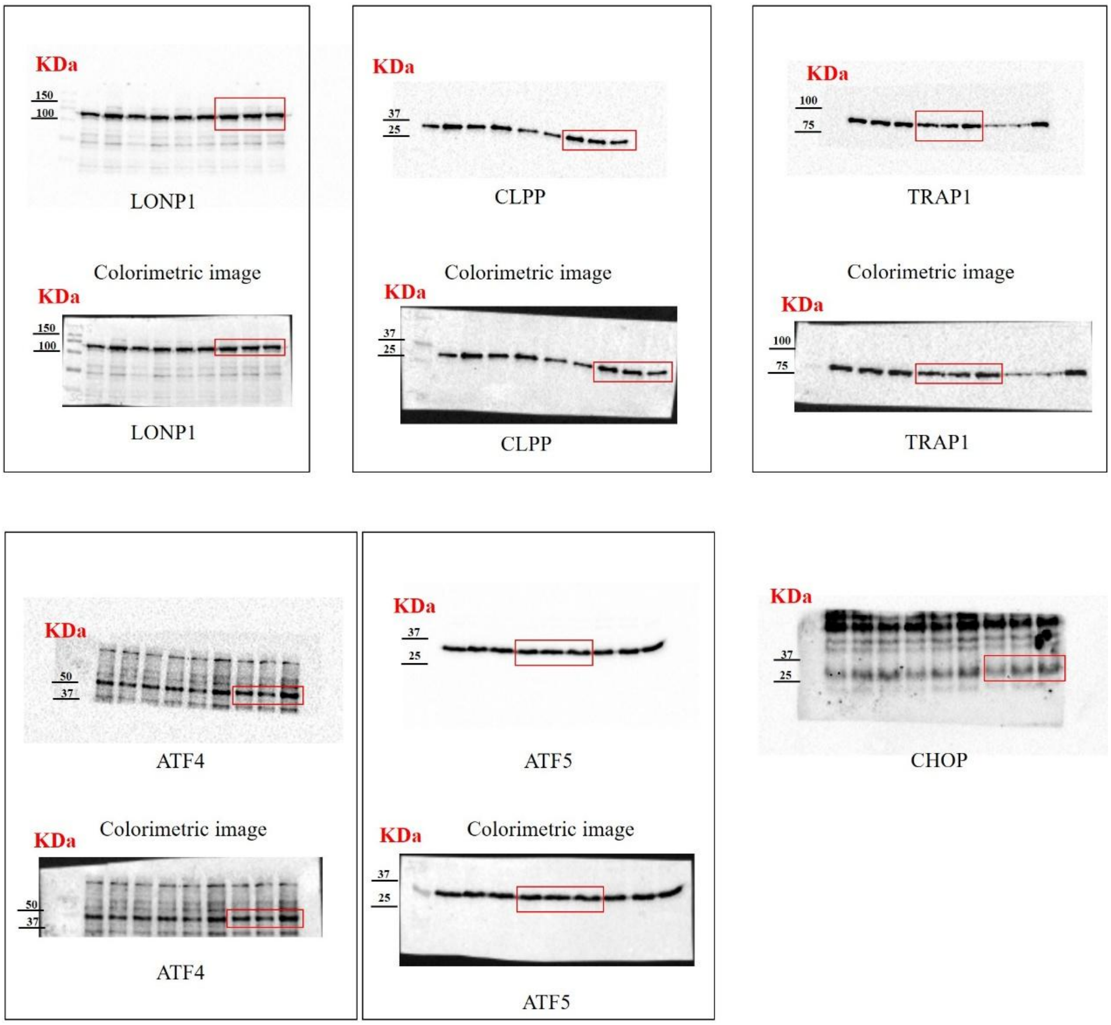

Figure S5. Uncropped Western blot images

Figure 6B

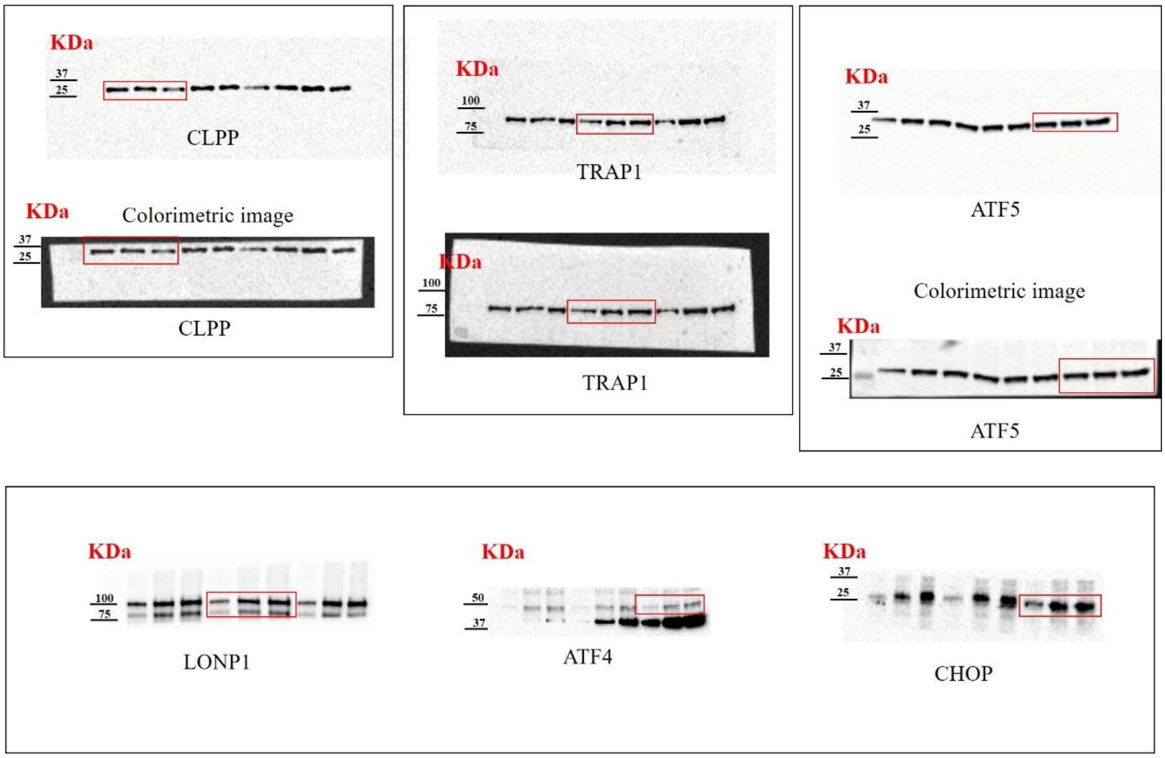

Figure S6. Uncropped Western blot images

Figure 6C

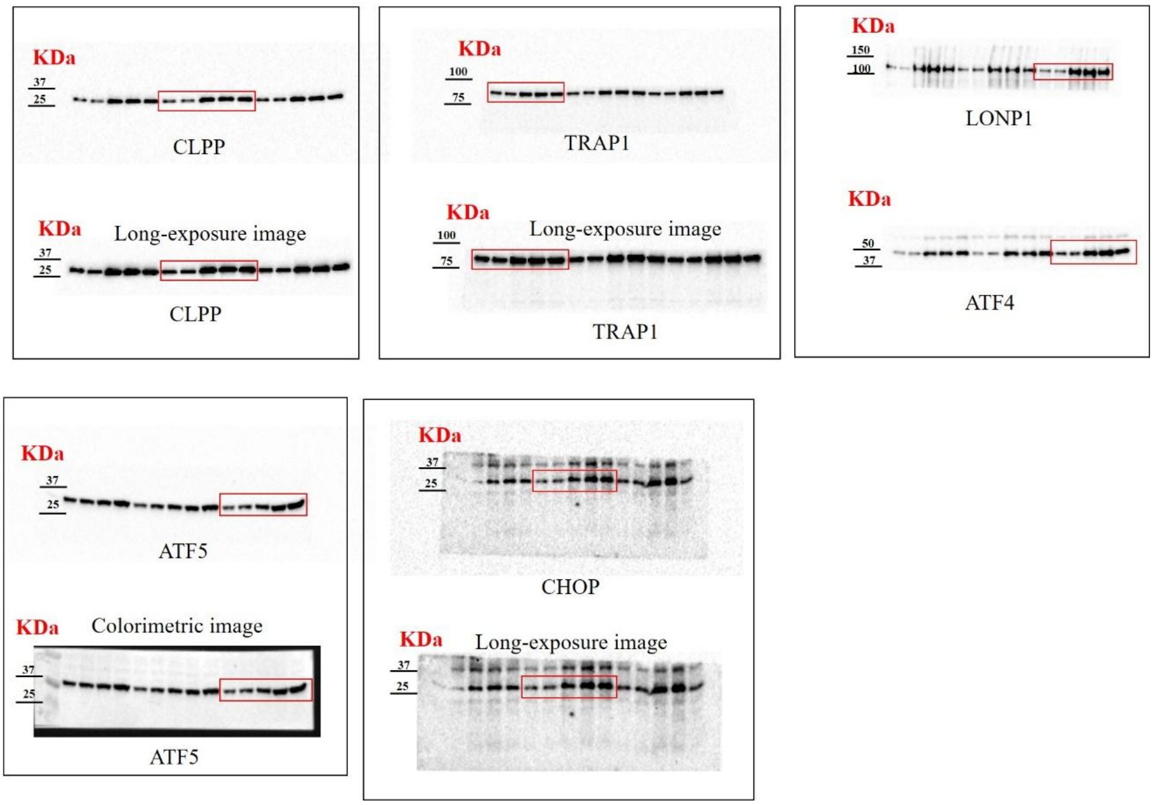

Figure S7. Uncropped Western blot images

Figure 6D

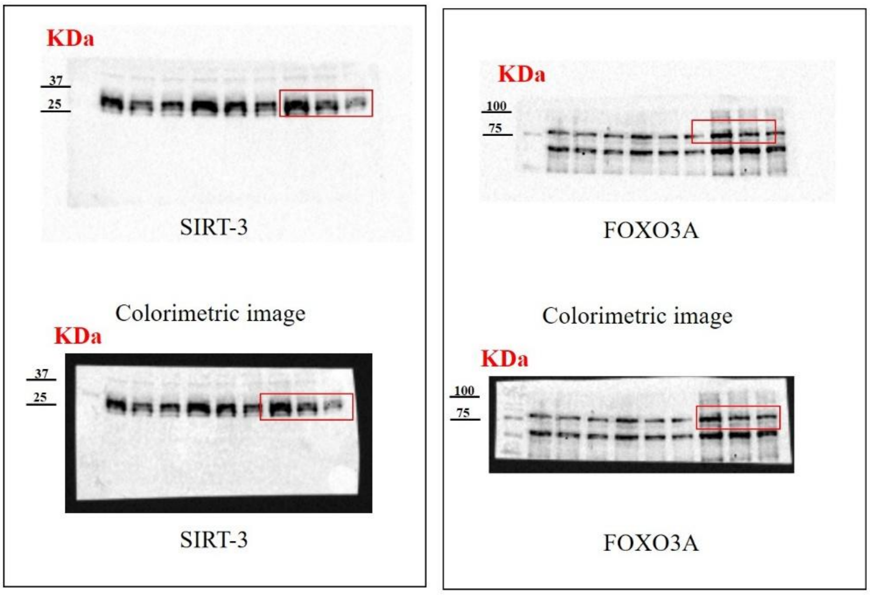

Figure 6E

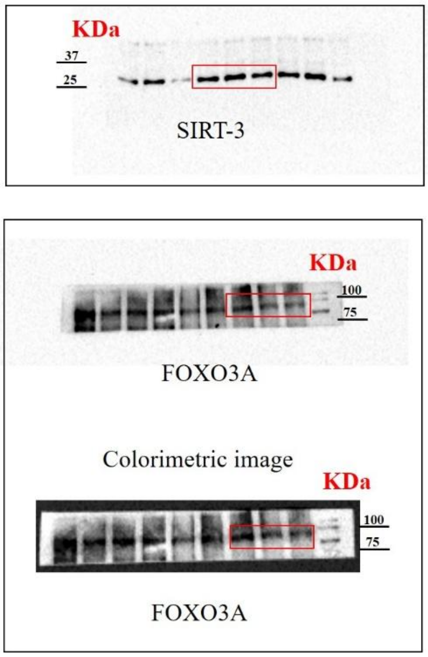

Figure 6F

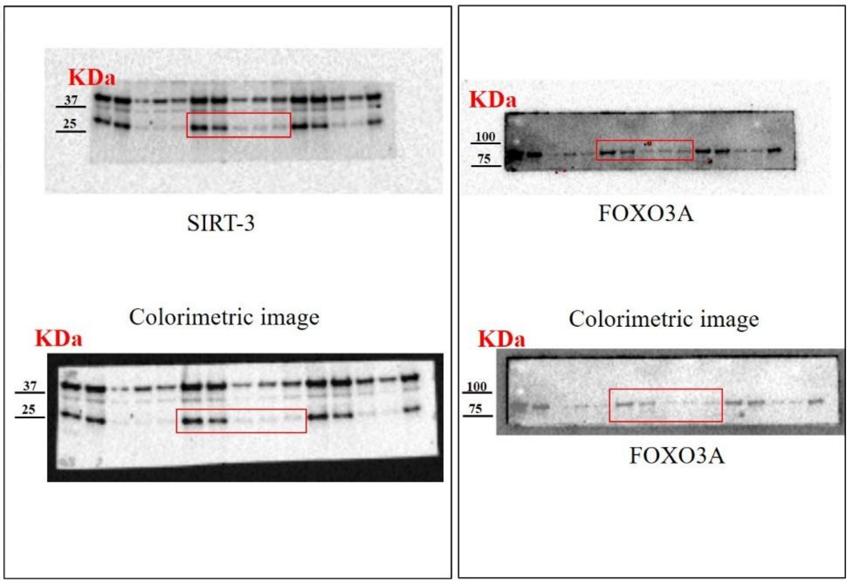

Figure S8. Uncropped Western blot images

Figure 7A

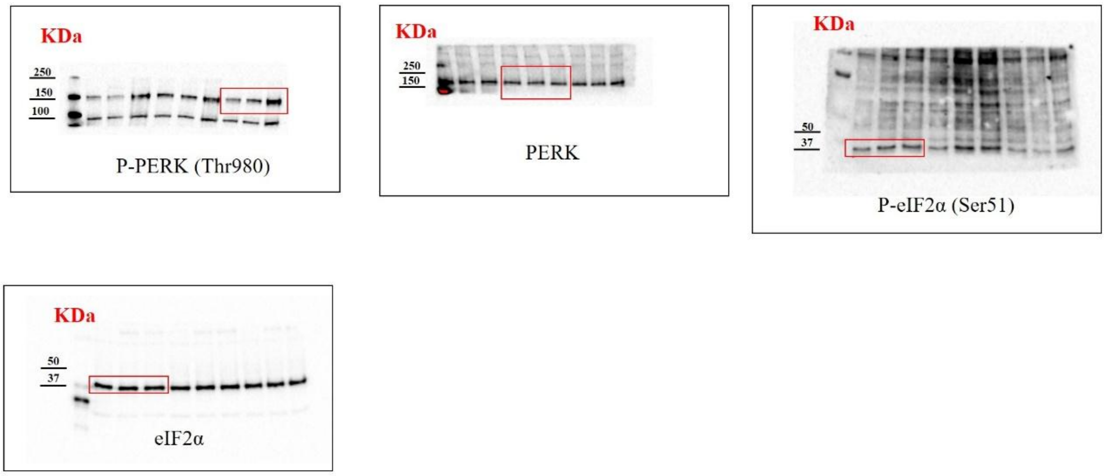

Figure 7B

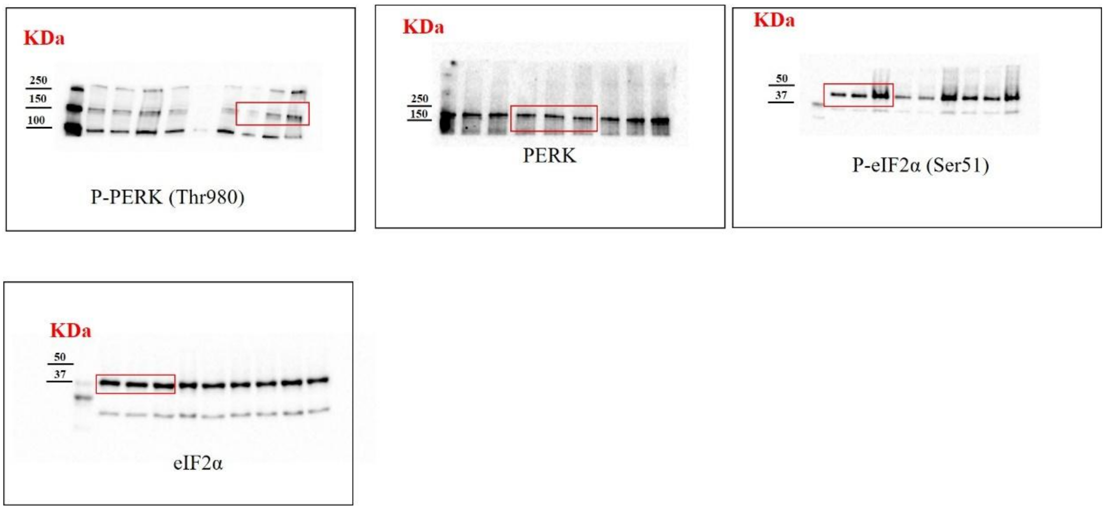

Figure S9. Uncropped Western blot images

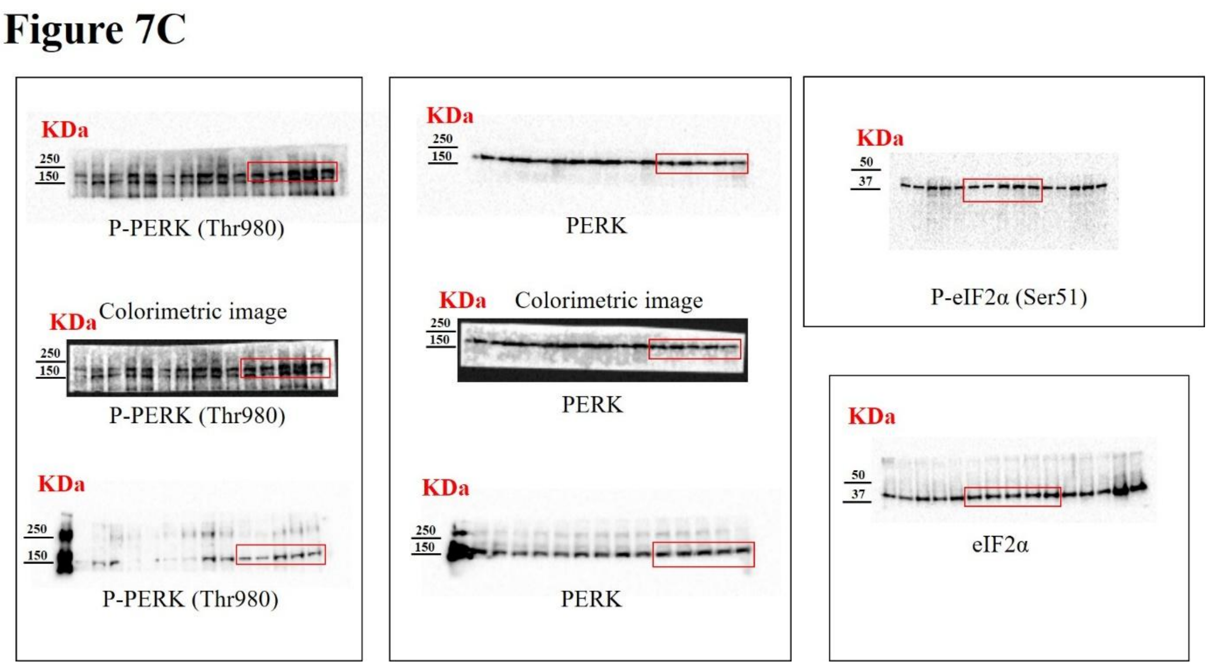

Figure S10. Uncropped Western blot images

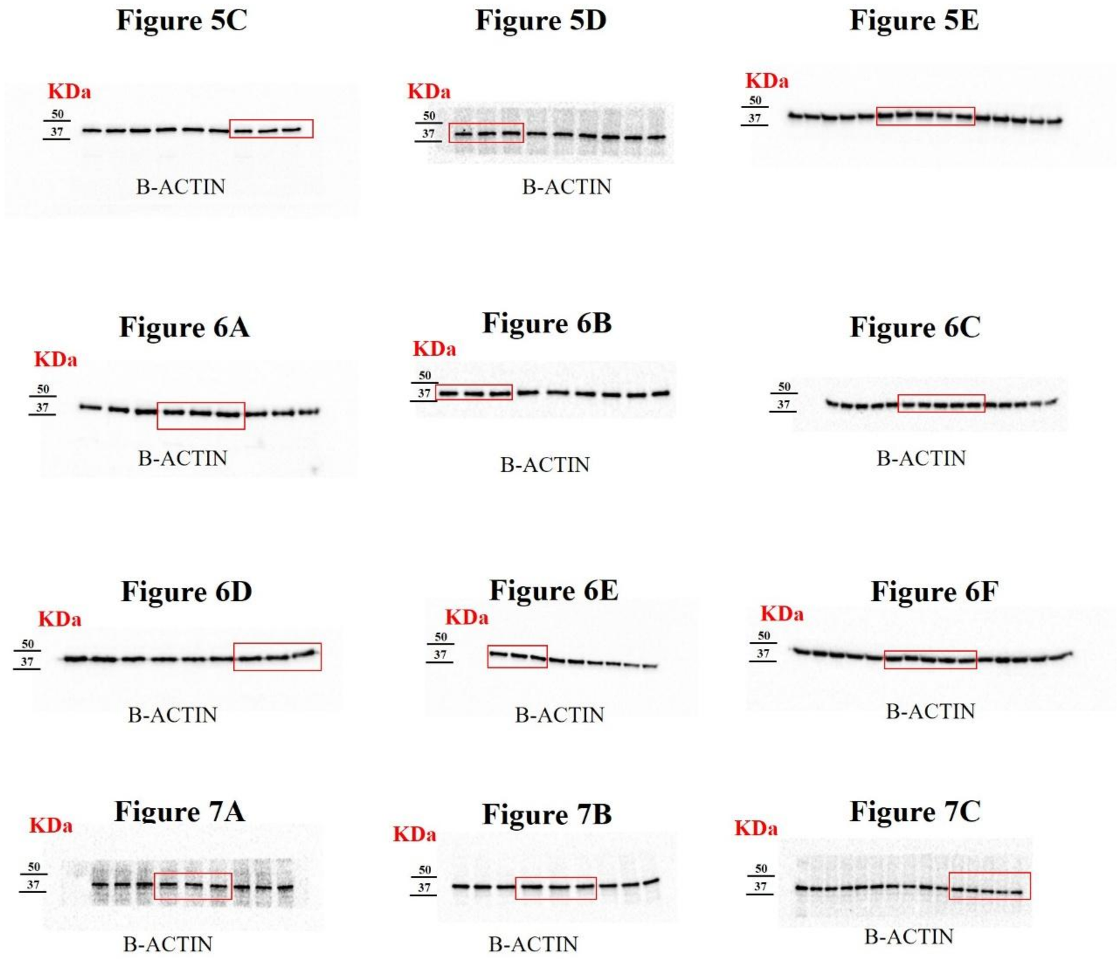

Figure S11. Uncropped Western blot images
